# TIM-3, LAG-3, or 2B4 gene disruptions increase the anti-tumor response of engineered T cells

**DOI:** 10.3389/fimmu.2024.1315283

**Published:** 2024-02-29

**Authors:** Beatrice Claudia Cianciotti, Zulma Irene Magnani, Alessia Ugolini, Barbara Camisa, Ivan Merelli, Valentina Vavassori, Alessia Potenza, Antonio Imparato, Francesco Manfredi, Danilo Abbati, Laura Perani, Antonello Spinelli, Eric Shifrut, Fabio Ciceri, Luca Vago, Raffaella Di Micco, Luigi Naldini, Pietro Genovese, Eliana Ruggiero, Chiara Bonini

**Affiliations:** ^1^ Experimental Hematology Unit, IRCCS San Raffaele Scientific Institute, Milan, Italy; ^2^ Innovative Immunotherapies Unit, IRCCS San Raffaele Scientific Institute, Milan, Italy; ^3^ Institute for Biomedical Technologies, National Research Council, Segrate, Italy; ^4^ Gene Transfer Technologies and New Gene Therapy Strategies Unit, San Raffaele Telethon Institute for Gene Therapy (SR-TIGET), IRCCS San Raffaele Scientific Institute, Milan, Italy; ^5^ Experimental Imaging Centre, IRCCS San Raffaele Scientific Institute, Milan, Italy; ^6^ The School of Neurobiology, Biochemistry and Biophysics, The George S. Wise Faculty of Life Sciences, Tel Aviv University, Tel Aviv, Israel; ^7^ Department of Pathology, Faculty of Medicine, Tel Aviv University, Tel Aviv, Israel; ^8^ Dotan Center for Advanced Therapies, Tel Aviv Sourasky Medical Center, Tel Aviv, Israel; ^9^ Hematology and Bone Marrow Transplantation Unit, IRCCS San Raffaele Scientific Institute, Milan, Italy; ^10^ Università Vita-Salute San Raffaele, Milan, Italy; ^11^ Unit of Immunogenetics, Leukemia Genomics and Immunobiology, IRCCS San Raffaele Scientific Institute, Milan, Italy; ^12^ San Raffaele Telethon Institute for Gene Therapy (SR-Tiget), IRCCS San Raffaele Scientific Institute, Milan, Italy; ^13^ Gene Therapy Program, Dana-Farber/Boston Children’s Cancer and Blood Disorders Center, Department of Pediatric Oncology, Harvard Medical School, Boston, MA, United States

**Keywords:** TCR - T cell receptor, adoptive T cell immunotherapy, inhibitory receptor, genome editing, CRISPR/Cas9

## Abstract

**Background:**

In adoptive T cell therapy, the long term therapeutic benefits in patients treated with engineered tumor specific T cells are limited by the lack of long term persistence of the infused cellular products and by the immunosuppressive mechanisms active in the tumor microenvironment. Exhausted T cells infiltrating the tumor are characterized by loss of effector functions triggered by multiple inhibitory receptors (IRs). In patients, IR blockade reverts T cell exhaustion but has low selectivity, potentially unleashing autoreactive clones and resulting in clinical autoimmune side effects. Furthermore, loss of long term protective immunity in cell therapy has been ascribed to the effector memory phenotype of the infused cells.

**Methods:**

We simultaneously redirected T cell specificity towards the NY-ESO-1 antigen via TCR gene editing (TCR_ED_) and permanently disrupted *LAG3*, *TIM-3* or *2B4* genes (IR_KO_) via CRISPR/Cas9 in a protocol to expand early differentiated long-living memory stem T cells. The effector functions of the TCR_ED_-IR_KO_ and IR competent (TCR_ED_-IR_COMP_) cells were tested in short-term co-culture assays and under a chronic stimulation setting *in vitro*. Finally, the therapeutic efficacy of the developed cellular products were evaluated in multiple myeloma xenograft models.

**Results:**

We show that upon chronic stimulation, TCR_ED_-IR_KO_ cells are superior to TCR_ED_-IR_COMP_ cells in resisting functional exhaustion through different mechanisms and efficiently eliminate cancer cells upon tumor re-challenge *in vivo*. Our data indicate that TIM-3 and 2B4-disruption preserve T-cell degranulation capacity, while LAG-3 disruption prevents the upregulation of additional inhibitory receptors in T cells.

**Conclusion:**

These results highlight that TIM-3, LAG-3, and 2B4 disruptions increase the therapeutic benefit of tumor specific cellular products and suggest distinct, non-redundant roles for IRs in anti-tumor responses.

## Highlights

- T cell exhaustion is a dysfunctional status dampening T cell effector functions and is driven by multiple inhibitory receptors. In this study we show that the disruption of three major inhibitory receptors (TIM-3 LAG-3 and 2B4) increases the antitumor activity of antigen-specific T cells. Our study will be pivotal in designing new tailored and more effective cancer immunotherapy approaches.

## Introduction

Adoptive T cell therapy (ACT) represents one of the most recent advances in biomedical research, and largely relies on the ability of T lymphocytes to recognize and kill their targets and to persist as memory cells. The transfer of genes encoding for high-affinity tumor-reactive T cell receptor (TCR) or chimeric antigen receptors (CAR) efficiently redirect T cell specificity towards tumor-associated and tumor-specific antigens (1–8). These living drugs display a number of distinctive qualities, compared to conventional chemical compounds, including the ability to respond to their target through expansion and contraction cycles and the capacity to persist and mediate long-term clinical effects (9). The efficacy of ACT largely relies on high-avidity cellular products. The application of genome editing technologies to T cells opened up a wide range of therapeutic opportunities for ACT. For instance, TCR gene editing allows to completely redirect T cell specificity, thus maximizing the tumor-specific TCR expression and the overall avidity and specificity of the cellular products (10, 11).

Several preclinical and clinical observations indicate that the ability of ACT to mediate prolonged clinical responses is associated with the long-term persistence of the infused T-cell products (12). Accordingly, manufacturing protocols favoring the generation of T-cell products endowed with an early differentiation, central memory T cells/stem cell memory T cells (T_CM_/T_SCM_) functional phenotype display superior anti-tumor activity, proliferation capacity, and long-term survival when compared to effectors (13). However, in cancer, chronic antigen stimulation and signals from the immunosuppressive tumor microenvironment cooperate in inducing T cell exhaustion, a dysfunctional differentiation status, that reduces and, in some cases, nullifies the therapeutic effect of ACT. While from an evolutionary perspective T cell exhaustion is beneficial to dampen excessive tissue damage, in cancer it represents a major immune evasion mechanism. Exhausted T cells display sustained upregulation of inhibitory receptors (IRs), including PD-1, CTLA-4, LAG-3, TIM-3, 2B4, CD39, CD160, BTLA and TIGIT, in the absence of co-stimulatory receptors. IR triggering results in a progressive and hierarchical loss of effector functions, desensitization of TCR, metabolic deregulation, altered expression of transcription factors required for effector functions and failure to acquire a memory phenotype (14–16).

Immune checkpoint blockade (ICB) reinvigorates exhausted cells and reactivates protective tumor-specific T cell responses (17, 18) and, although the clinical use of ICBs has shown considerable benefits, some drawbacks limit its broad applicability. First, the efficacy of ICBs is restricted to immunogenic tumors and even within those cancer types, only 20% of patients achieves durable responses as a substantial fraction of treated patients shows lack of initial responses (i.e. primary resistance) or develops failure of response overtime (i.e. acquired resistance) (19). Several mechanisms have been ascribed to ICB failure. Among them, the upregulation of alternative immune checkpoints by TILs upon ICB therapies in hot-immunogenic tumors suggests a non-redundant role of IRs (20, 21). Furthermore, the widespread action of ICBs on the entire T-cell repertoire is responsible for several immune-related adverse events (irAEs), reported in 90% of treated patients, with incidence of severe (grade 3-5) irAEs ranging from 20 to 60% (19, 22). Strategies to tailor inhibitory receptor disruption in tumor-specific cellular products have been proposed to overcome these limitations, but are mainly focused on PD-1- or CTLA-4- disrupted cells tested in short-term anti-tumor responses (23–31). Here, we selected TIM-3, LAG-3 and 2B4, three inhibitory receptors frequently expressed by T cells in several cancer types and involved in resistance to immunotherapy (32, 33), and we exploited the multiplexicity of CRISPR/Cas9 with lentiviral vectors to simultaneously redirect T cell specificity and disrupt genes encoding for TIM-3, LAG-3, or 2B4, within a protocol able to expand long-living early differentiated T_SCM_ (34). With this approach, we investigated the relative contribution of distinct IR knocked-out in tumor specific cellular products chronically challenged by multiple myeloma cells.

## Methods

### Primary T cells and cell lines

Peripheral blood mononuclear cells (PBMCs) were collected from healthy donors, after written informed consent, according to the San Raffaele Scientific Institutional Ethical Committee guidelines. The PBMCs were then isolated by Ficoll-Hypaque gradient separation (Lymphoprep; Fresenius) and the T cells were cultured as previously described (34). Briefly, PBMC harvested from healthy donors were activated using anti-CD3/anti-CD28-coated magnetic beads (ClinExVivo CD3/CD28; Invitrogen) and maintained at a concentration of 106 cells/mL in complete X-vivo supplemented with IL-7 and IL-15 (5ng/mL each). Two days after stimulation, T cells were electroporated with ribonucleoprotein (RNP) complexes (consisting of purified Spy Cas9 nuclease duplexed with synthetic gRNAs specific for the indicated genes) simultaneously using the Lonza Nucleofector 4D Electroporation System. The day after, T cells were transduced with a lentiviral vector (LV) encoding for the NY-ESO1-specific TCR. At day 6 post stimulation, beads were removed from culture and at day 18 tested for phenotype and function.

Myeloma cell lines (MM.1s and U266) were grown with RPMI 1640 supplemented with 10% fetal bovine serum (FBS), 1% penicillin/streptomycin and 1% glutamine (Lonza). Adherent MM.1s were detached using TrypLE Express enzyme (Gibco). To obtain HLA-A2^pos^ NY-ESO-1^pos^ luciferase^pos^ MM1.s cells, wild-type MM1.s were transduced with 3 different LVs encoding for HLA-A2, NY-ESO-1 and ΔLNGFR, luciferase and GFP. Transduced cells were FACS-sorted to obtain a pure population (> 98%) of HLA-A2^pos^ NY-ESO-1^pos^ luciferase^pos^ cells (MM1.s A2^pos^ ESO-1^pos^). HLA-A2, ΔLNGFR, and GFP transgene expressions were quantified by flow cytometry. In selected experiments, for IR ligand evaluations, cell lines were stimulated with IFNγ (600IU/mL) for 48 hours. Galectin-9 and HMGB1 secretions in cell culture medium were quantified using Galectin-9 ELISA kit (RayBiotech) or HMGB1 ELISA kit (IBL International) according to the manufacturer’s instructions. CD48 and HLA- DR expressions were quantified by flow cytometry upon staining with fluorochrome conjugated monoclonal antibodies. Cells were counted every 3-4 days by Trypan blue dye exclusion.

#### IR disruption and TCR gene editing

The gRNAs sequences used for IR and TCR disruption were as follows: TRAC: 5’- GAGAATCAAAATCGGTGAAT**AGG**-3’; TIM-3: 5’- GAGTCACATTCTCTATGGTC**AGG**-3’; LAG-3: 5’- CACCGCGGCGCGGTACTCGC**CGG**-3’; 2B4: 5’- AGTTGAGAAACCCCGCCTAC**AGG**-3’. The PAM sequence is indicated in bold. Commercial crRNA (Alt-R^®^ CRISPR-Cas9 crRNA, IDT) and tracrRNA (Alt-R^®^ CRISPR-Cas9 tracrRNA, IDT) were mixed in equimolar concentrations and heated at 95°C for 5 min to form a duplex with a final 100 μM concentration. crRNA:tracrRNA duplex and Cas9 enzyme were assembled in a 120:104 pmol ratio for 20 minutes at RT according to the manufacturer’s instructions. For each RNP targeting a single gene, 1 uL of Enhancer (100 uM; Alt-R^®^ Electroporation Enhancer, IDT) was added to the reaction. Stimulated human CD3^+^ cells were electroporated 48 hours after stimulation. Two million cells/condition were resuspended in P3 primary buffer (Lonza) and assembled RNPs targeting one or more coding sequences of selected genes were simultaneously added to the cell suspension. Cells were electroporated using Nucleofector 4D (Lonza) according to manufacturer’s instructions; 24 hours later, the electroporated cells were counted and transduced with a previously validated lentiviral vector encoding for an HLA-A2 restricted NY-ESO-1_157-165_ specific TCR. The expression of the tumor-specific TCR was evaluated by flow cytometry through anti-Vβ13.1 antibody (FITC, Beckman Coulter) or with an APC-conjugated dextramer (Immudex).

### Gene disruption efficiency analysis

Targeted T lymphocytes were collected at least one week after genomic manipulation to evaluate the frequency of non-homologous end joining (NHEJ) at each targeted genomic locus. DNA was extracted through QIAamp DNA Mini or Micro kits (Qiagen), depending on the number of cells and according to the manufacturer’s instructions. NHEJ assessment in manipulated cells was performed using ddPCR NHEJ custom Genome Edit Detection Assays (Bio-Rad) to amplify the TIM-3, LAG-3, 2B4 and TRAC targeted loci, according to the manufacturer’s instructions, with the only exception being the time and temperature of annealing/extension, 3 minutes and 59.4°C, respectively. Data were acquired using QuantaSoft Software and analyzed with QuantaSoft Analysis Pro Software.

#### *In vitro* co-culture assays

Killing assays were performed by co-colturing multiple myeloma cells for 3 days with TCR_ED_-IR_KO_ or TCR_ED_-IR_COMP_ T cells at decreasing effector:target (E:T) ratio. Wild type HLA-A2^neg^NY-ESO-1^neg^ MM.1s cell line and unedited, untransduced T lymphocytes (UT) were used as controls. After three days, cells were analyzed by flow cytometry. U266 cells were selected as CD138^+^ cells; HLA-A2^pos^NY-ESO-1^pos^ MM1.s were selected as GFP^+^ cells, whereas wild type MM.1s were selected as CD38^+^ cells. The number of residual target cells were counted according to the number of cells acquired at flow cytometry and the total well volume.

Cytokine production was analyzed 24 hours after antigen-specific stimulation. A panel of 13 different cytokines was quantified in supernatants of co-colture assays using LEGENDplex human Th panel (13-plex, Biolegend) according to the manufacturer’s instruction, and the data were analyzed using LEGENDplex data analysis software (Vigentech).

For degranulation assays, target and effector cells were co-coltured for 6 hrs in a 1:1 E:T ratio and CD107a^+^ CD3^+^ T cells were quantified by flow cytometry. Medium, HLA-A2^neg^NY-ESO-1^neg^ MM1.s wild-type cells and unedited, untransduced T lymphocytes (UT) were used as negative controls. T cells stimulated with 50 ng/ml PMA (Sigma-Aldrich) and 1mg/mL ionomycin (Sigma-Aldrich) were used as positive control. In chronic stimulation experiments, effector cells were stimulated daily with target cells in a 10:1 E:T ratio. After 15-21 days, effector and target cells were co-coltured to evaluate killing, degranulation and cytokine production. In all functional assays performed after chronic stimulation, effector:target ratio were calculated considering the viable number of CD3^+^ T cells at end of chronic stimulation. Absence of residual target cells after chronic stimulation was ensured by flow cytometry.

### RNA-seq

Twenty-four hours after co-culture with HLA-A2^pos^ NY-ESO-1^pos^ MM1.s cell line (effector: target ratio = 1:1), T cells were collected for RNA extraction. Total RNA was extracted with RNeasy mini kit (QIAGEN) according to manufacturer’s instructions. RNA was quantified with The Qubit 2.0 fluorometer (ThermoFisher) and its quality was assessed by Agilent RNA ScreenTape system (Agilent). RNA library preparations and sequencing reactions were conducted at GENEWIZ, LLC. (South Plainfield, NJ, USA). SMART-Seq v4 Ultra Low Input Kit for Sequencing was used for full-length cDNA synthesis and amplification (Clontech, Mountain View, CA) and Illumina Nextera XT library was used for sequencing library preparation. Briefly, cDNA was fragmented and adaptor was added using Transposase, followed by limited-cycle PCR to enrich and add index to the cDNA fragments. The final library was assessed with Agilent TapeStation. The sequencing libraries were multiplexed and clustered on a flowcell. After clustering, the flowcell was loaded on the Illumina HiSeq instrument according to manufacturer’s instructions. The samples were sequenced using a 2x150 Paired End (PE) configuration. Image analysis and base calling were conducted by the HiSeq Control Software (HCS). Raw sequence data (.bcl files) generated from Illumina HiSeq was converted into fastq files and de-multiplexed using Illumina’s bcl2fastq 2.17 software. One mis-match was allowed for index sequence identification.

### RNA sequencing data analysis

Raw single-end reads quality control was determined using FastQC tool (http://www.bioinformatics.babraham.ac.uk/projects/fastqc) and read trimming was performed using Trim Galore software (https://doi.org/10.5281/zenodo.5127899) to remove residual adapters and low-quality sequences. Trimmed reads were aligned against the human reference genome (GRCh38) using STAR (35) with standard input parameters and only uniquely mapped reads were considered for downstream analyses. Reads were assigned to genes with the featureCounts tool (36) using the GENCODE primary assembly v.35 gene transfer file (GTF) as reference annotation for the genomic features. Gene count matrices were then processed by the R/Bioconductor differential gene expression analysis packages DESeq2 (37) following standard workflows. In particular, a paired analysis was set up, modelling gene counts using the following design formula: ~donor + condition. Genes with adjusted p-values less than 0.1 were considered differentially expressed.

A functional enrichment analysis was performed on the lists of significantly upregulated and downregulated DEGs. Enrichment analysis was performed considering the MSigDB database (C5: Ontology Gene Sets) using the enricher function from the R/Bioconductor package clusterProfiler (38) (v 3.8.1). Enriched terms with an adjusted p-value < 0.05 were considered statistically significant. Volcano plots were generated using the R package ggplot2 (https://ggplot2.tidyverse.org) and have been used to display RNA-seq results plotting the statistical significance (adjusted p-value) versus the magnitude of change (fold change). Heatmaps were generated using the R package pheatmap (https://CRAN.R-project.org/package=pheatmap). UpSet analysis was performed using UpSetR shiny app (39).

### *In vivo* experiments

The protocol was approved by the Institutional Animal Care and Use Committee (IACUC). For the U266 mouse models, 6-8 weeks old female NOD/Scid gamma mice (NSG, Charles-River Italia) received sub-lethal total body irradiation (150 rads) and were then infused intravenously with 10x10^6^ luciferase^pos^ U266 cells (U266 dluc). Tumor growth was monitored by total body bioluminescent imaging (BLI) with IVIS SpectrumCT System. Briefly, mice were injected intraperitoneally with 150mg luciferin/kg 10 minutes before BLI. BLI image analysis was performed by measuring the total flux (photons/seconds) within regions with lesions. Images were acquired and analyzed with Living Image 4.4. Upon specific tumor engraftment (according to the experimental settings), mice were treated with different doses of engineered or untransduced T lymphocytes. High-tumor burden was defined as total flux > 10^6^. Human chimerism and activation of T cells on peripheral blood were assessed weekly by flow cytometry. Mice were monitored weekly and in the presence of limping or weight loss greater than 5%, they were sacrificed. At sacrifice, spleen and bone marrow were harvested and extensive phenotypic, activation and exhaustion analysis of human T cells was performed by flow cytometry. In the U266 re-challenge mouse model, 10x10^6^ U266 dluc were infused intravenously in mice that showed an anti-tumor response at day 14 after T cell treatment.

### Multiparametric flow cytometry

Live/Dead Fixable Violet Dead cell stain kit or DAPI were used to exclude dead cells prior to surface staining (Thermo-Fisher). For intracellular staining, anti-CD107a antibody (eBioscience) was added to cell culture. Cells were stained with surface antibodies, fixed and permeabilized with FIX & PERM Cell Fixation & Permeabilization Kit (ThermoFisher), according to the manufacturer’s instructions.

For phospho-flow experiments, T cells were stimulated with 5mM of H_2_O_2_ for 8 minutes. After stimulation, cells were fixed with 200μL of Fixation Buffer (BD CytofixTM Buffer) at 37°C for 10 minutes. Fixed cells were then washed in PBS with 1% FBS and permeabilized with Permeabilization Buffer (BD PhosflowTM Perm Buffer III) on ice for 45 minutes. Permeabilized cells were then washed, resuspended in PBS with 1% FBS and stained with Pacific Blue-conjugated anti-phospho-ERK and PerCP-conjugated anti-CD3 antibodies for 45 minutes on ice. After staining, cells were washed in PBS with 1% FBS and samples analyzed by BD FACS Canto II flow cytometer.

For *in vivo* experiments, 50-100 μL of whole blood were stained with fluorochrome conjugated monoclonal antibody specific for mouse CD45, human CD45, 2B4, CD3, CD8, CTLA-4, PD-1, TIM-3, LAG-3, HLA-DR, CD45RA, CD62L. After surface staining, red blood cells were lysed with ACK (Ammonium-Chloride-Potassium) buffer for 10 minutes at room temperature. Human T cells were counted on peripheral blood using Flow Count fluorescent beads (Beckman Coulter), according to manufacturer’s instruction. For phenotype analysis in chronic antigen stimulation experiments, stimulated T cells were stained with fluorochrome conjugated monoclonal antibodies specific for CD3, CD8, CD4, LAG-3, CD45RA, CD62L, HLA-DR, CTLA4, PD-1, TIM-3, 2B4, TIGIT, CD160, CD39.

For each experiment, dead cells were excluded by DAPI positive staining. Data were acquired using a BD FACS Canto II, LSR Fortessa or Symphony (BD Biosciences) and analyzed with FlowJo version 10 software (TreeStar). Memory subsets were identified using CD45RA and CD62L surface markers, as previously described (40). Briefly, stem cell memory cells (T_SCM_) were gated as CD45RA^+^ CD62L^+^ T cells; central memory cells (T_CM_) were gated as CD45RA^-^ CD62L^+^ T cells; effector memory cells (T_EM_) were gated as CD45RA^-^ CD62L^-^ T cells; CD45RA-expressing effector memory cells (T_EMRA_) were gated as CD45RA^+^ CD62L^-^ cells.

All antibodies used for flow cytometry are listed in **Supplementary Table 1**.

### High dimensional flow cytometry data analysis

Flow Cytometry Standard (FCS) files were analyzed using FlowJo software (ver.10) to remove doublets and dead cells and to isolate CD3^+^ T cells. Down sampling was performed to export 3000 CD3+ events/samples. Subsequent clustering analysis was performed using a public available pipeline (41–43). The k value was set to 30 for clustering analysis.

### Statistical analysis

Statistical analyses were performed with Prism 9 (GraphPad Software). The Mann-Whitney test was used when comparing two independent groups. A two-way ANOVA was used when comparing variables across two or more normally distributed subsets. Linear regression analysis was used to model the linear relationships between analyzed variables. For all comparisons, two-sided P values were used and p < 0.05 was considered statistically significant.

## Results

### CRISPR-Cas9-mediated TIM-3, LAG-3 or 2B4 disruption does not alter the expansion capacity nor the differentiation phenotype of TCR_ED_ T cells

**Figure 1:**
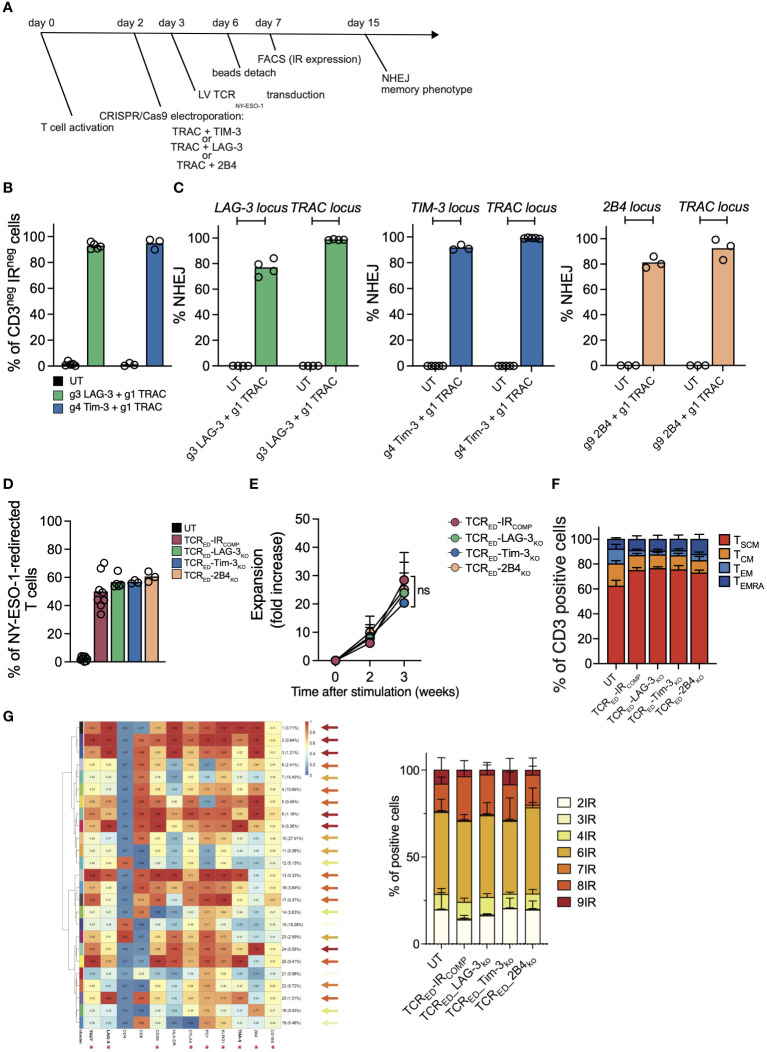
CRISPR-Cas9-mediated TIM-3, LAG-3 or 2B4 disruption does not alter the expansion capacity nor the differentiation phenotype of TCR_ED_ T cells. **(A)** Schematic representation of the protocol for the generation of TIM-3-, LAG-3, or 2B4-disrupted TCR-edited (TCR_ED_) T cells. **(B)** Percentages of CD3^neg^LAG-3^neg^ (green bar; N=5) or CD3^neg^TIM-3^neg^ (blue bar; N=3) live cells measured by flow cytometry in T cells electroporated with CRISPR/Cas9 targeting LAG-3 or TIM-3 in combination with CRISPR/Cas9 targeting TRAC. Unmanipulated cells (UT, black bars) are shown as control. **(C)** Percentages of NHEJ at *LAG-3* (green bar; N=4), *TIM-3* (blue bar; N=3/5), and *2B4* (light orange bar; N=3) loci in UT cells (black bar; N=3/5) and in T cells electroporated with CRISPR/Cas9 targeting the specific inhibitory receptor and the *TRAC* locus. **(D)** Frequency of cells redirected against NY-ESO-1 upon lentiviral transduction of unmanipulated cells (UT, black bar; N=8) or in T cells electroporated with CRISPR/Cas9 targeting *TRAC* alone (TCR_ED-_IR_COMP_, red bar; N=8) or in combination with gRNAs targeting *LAG-3* (TCR_ED_-LAG-3_KO_, green bar; N=5), *TIM3* (TCR_ED_-TIM-3_KO_, blue bar; N=3) or *2B4* (TCR_ED_-2B4_KO_, light orange bar; N=3). Transduction efficiency was measured by flow cytometry upon HLA-A*02:01-NY-ESO-1_157-165_ dextramer staining. Fold increase **(E)** and memory phenotype **(F)** of inhibitory receptor (IR) competent (TCR_ED_-IR_COMP;_ N=5), TCR_ED_-LAG-3_KO_ (N=4), TCR_ED_-TIM-3_KO_ (N=3) and TCR_ED_-2B4_KO_ (N=3) T cells at day +21. g1, g3, g4, g9 refer to gRNAs designed to disrupt the indicated genes. **(G)** Meta-cluster composition of TCR_ED_ or TCR_ED_-IR_KO_ T cells (left). Arrows colors indicate the number of IR co-expressed in each cluster according to the color code indicated on the right. Percentages of cells co-expressing from 2 to 9 of the IRs marked with an asterisk, in TCR_ED_-IR_COMP_ (N=3) or TCR_ED_-IR_KO_ (N=3) cells are shown on the right. Data are shown as mean ± SEM of 3-8 biological replicates; ns= not statistically significant.

To generate T_SCM_ redirected against tumor antigens and resistant to exhaustion signals, we combined the multiplexing capacity of CRISPR/Cas9 with the efficiency of lentiviral vectors for the simultaneous TCR genetic redirection and IR genetic ablation following a protocol that favors the expansion of early differentiated T cells (**Figure 1A**). We designed and tested multiple sgRNAs targeting *TRAC*, *TIM-3 (HAVCR2)*, *LAG-3* and *2B4 (CD244)* loci and selected those with the highest gene disruption efficiencies (**Supplementary Figure 1A**). CRISPR/Cas9-RNPs targeting *TRAC* and one selected IR locus (*TIM-3, LAG-3* or *2B4*) were simultaneously delivered by electroporation in activated T cells. Since CD3 requires an intact TCR to translocate to cell surface while TIM-3 and LAG-3 are expressed at moderate to high levels by more than 80% of activated CD3^+^ T cells in the first 6 days after stimulation (**Supplementary Figures 1B, C**), we quantified the efficiency of TRAC, TIM-3 and LAG-3 disruption by flow cytometry. Upon double gene disruption, we observed a median of 94.7% and 92.7% of CD3_NEG_-TIM-3_NEG_ and CD3_NEG_-LAG-3_NEG_ cells respectively (**Figure 1B**).

By quantifying NHEJ events at each targeted locus with ddPCR, we observed a median efficiency of 2B4 genetic disruption of 81.53% and we confirmed high efficiency of knock-out at the *LAG-3*, *TIM3* loci (76.8% and 91.6% respectively) in double TRAC-IR knock-out cells (**Figure 1C**).

TCR_NEG_-IR_KO_ and TCR_NEG_ cells competent for IR expression (TCR_NEG_-IR_COMP_) were transduced with a lentiviral vector encoding for an HLA-A2-restricted-NY-ESO-1_157-165_ specific TCR (4) to obtain a pool of TCR edited cells (TCR_ED_-IR_COMP,_ TCR_ED_-LAG-3_KO_, TCR_ED_-TIM-3_KO_, TCR_ED_-2B4_KO_) specific for the same tumor antigen. As shown in **Figure 1D**, transduction efficiency, measured by flow cytometry, was higher than 50% in all cellular products. Of note, TCR_ED_-IR_COMP_ and TCR_ED_-IR_KO_ T cells displayed similar expansion kinetics (**Figure 1E**) and were equally enriched in early differentiated memory cells at the end of the expansion (**Figure 1F**). Gene modifications (IR disruption and NY-ESO-1 specific TCR expression) and memory phenotype were similar in CD4^+^ and CD8^+^ T-cell subsets present in our cellular products (**Supplementary Figures 2A–D**). In addition, exploiting unsupervised high-dimensional analysis we could assess the percentage of cells co-expressing from 2 to 9 different IR among TIGIT, LAG-3, CD39, CTLA4, PD1, KLRG1, TIM3, 2B4, and CD160. Noticeably, no differences were observed in IR co-expression in our cellular products, with approximately 25% of cells expressing ≤ 4 IR (**Figure 1G**).

Overall, these data demonstrate that we can efficiently generate high numbers of early differentiated T cells redirected against a tumor antigen and permanently devoid of one inhibitory receptor. Importantly, we showed that the disruption of LAG-3, TIM-3 or 2B4 does not impair the expansion capacity nor the memory phenotype of edited CD4^+^ and CD8^+^ T cells *in vitro*.

### IR disruption increases the production of effector molecules in TCR gene edited T cells

**Figure 2:**
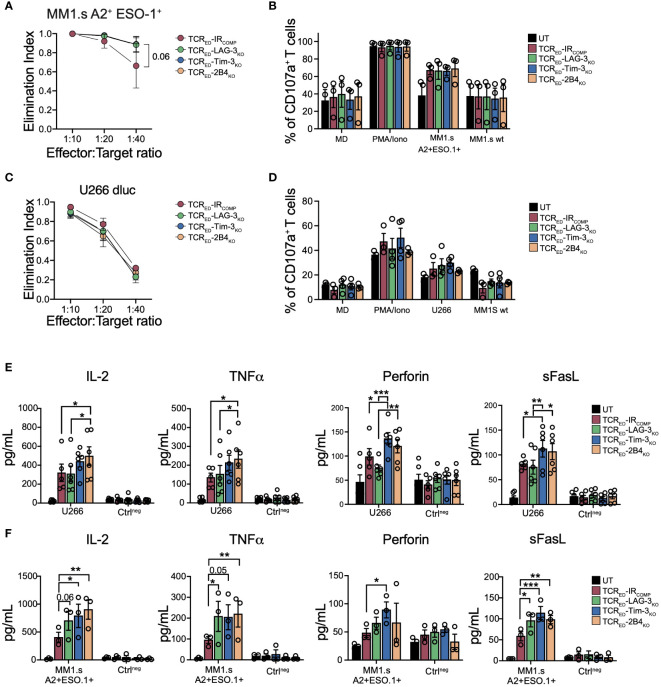
IR disruption increases the production of effector molecules in TCR gene edited T cells. Elimination index **(A, C)** and degranulation **(B, D)** of TCR_ED_-IR_COMP_ (red), TCR_ED_-LAG-3_KO_ (green), TCR_ED_-TIM-3_KO_ (blue) and TCR_ED_-2B4_KO_ (light orange) T cells co-cultured with U266 or HLA-A2^neg^ NY-ESO-1^neg^ MM1.s (MM1.s A2^+^ESO-1^+^) or HLA-A2^neg^ NY-ESO-1^neg^ MM1.s (MM1.s wt). In panel **(B, D)** only medium (MD) and PMA/Ionomycin (PMA/iono) were used as controls. Degranulation of untransduced (UT, black bars) cells is shown. **(E, F)** Quantification of IL-2, TNFα, perforin, and sFasL produced by TCR_ED_-IR_COMP_ (red bars), TCR_ED_-LAG-3_KO_ (green bars), TCR_ED_-TIM-3_KO_ (blue bars), and TCR_ED_-2B4_KO_ (light orange bars) T cells or UT T cells (black bars) upon exposure to HLA-A2^pos^ NY-ESO^pos^ U266 cells **(E)** or MM1.s A2^pos^ESO-1^pos^ cells **(F)**. Negative controls (Ctrl^neg^) indicate exposure of effector cells to medium or wild-type MM1.s cells. Data are shown as mean ± SEM of 3-6 biological replicates; **(A, B, F)** TCR_ED_-IR_COMP_ (N=3), TCR_ED_-LAG-3_KO_ (N=3), TCR_ED_-TIM-3_KO_ (N=3) and TCR_ED_-2B4_KO_ (N=3) UT (N=3); **(C, D)** TCR_ED_-IR_COMP_ (N=3), TCR_ED_-LAG-3_KO_ (N=4), TCR_ED_-TIM-3_KO_ (N=4) and TCR_ED_-2B4_KO_ (N=3) UT (N=4); **(E)** TCR_ED_-IR_COMP_ (N=5), TCR_ED_-IR_KO_ and UT (N=6)). *: p value <0.05; **: p value < 0.01; ***: p value < 0.001. Elimination index=1-[number of living target cells co-cultured with redirected T cells/number of living target cells co-cultured with UT cells].

Upon antigen recognition, cancer cells foster inhibitory signals through the upregulation of IR ligands and the pattern of IR engagement displays both inter-tumor and intra-tumor variability. To investigate if multiple myeloma cells engage TIM-3-, LAG-3- and 2B4-mediated pathways, we analyzed the expression of IR ligands in two multiple myeloma cell lines (U266 and MM1.s) in both resting conditions and in pro-inflammatory conditions. We quantified the release in cell culture medium of Gal-9 and HMGB1 (TIM-3 ligands) and of FGL-1 (LAG-3 ligand), the flipping of phosphatidyl-serin (TIM-3 triggering signal) in the outer layer of the cell membrane, the membrane bound Gal-9 and CEACAM (TIM-3 ligands) and the upregulation of MHC-II (LAG-3 ligand) and CD48 (2B4 ligand). Both MM1.s and U266 cell lines expressed all tested IR ligands, albeit at varying levels. Of note, the level of IR ligands remained stable upon IFN-γ exposure (**Supplementary Figures 3A, B**). To test the effect of TIM-3, LAG-3 and 2B4 disruption in tumor-specific T cells upon antigen recognition on cancer cells expressing IR ligands, we challenged TCR_ED_-IR_COMP_ and TCR_ED_-IR_KO_ T cells with either U266 (a cell line naturally expressing HLA-A2 and NY-ESO-1) or MM1.s transduced to express HLA-A2 and NY-ESO-1 antigen and luciferase (MM1.s A2^pos^ESO-1^pos^, **Supplementary Figures 3C, D**). In a short-term co-culture assay, we observed that the disruption of TIM-3, LAG-3, and 2B4 does not impact the killing efficiency nor degranulation ability of T cells (**Figures 2A–D**), with the exception of a slight increase in MM1.s A2^pos^ESO-1^pos^ killing observed in all TCR_ED_-IR_KO_ cells at limiting effector:target ratio. Interestingly, the analysis of secreted pro-inflammatory cytokines and effector molecules showed that TIM-3-, LAG-3-, and 2B4-disrupted TCR_ED_ T cells produce higher amounts of IL-2, TNFα, sFasL and perforin compared to TCR_ED_-IR_COMP_ T cells when tested with the two different multiple myeloma models (**Figures 2E, F** **;** **Supplementary Figure 4A**). TCR_ED_-IR_COMP_ and all TCR_ED_-IR_KO_ cells produced equal and high amount of IFNγ (**Supplementary Figure 4B**). These results indicate that the absence of TIM-3, LAG-3, or 2B4 in tumor-specific T cells does not affect their killing capacity nor degranulation ability while induces an increased production of pro-inflammatory cytokines and molecules.

### TCR_ED_-IR_KO_ cells display a unique transcriptional profile upon target recognition

**Figure 3:**
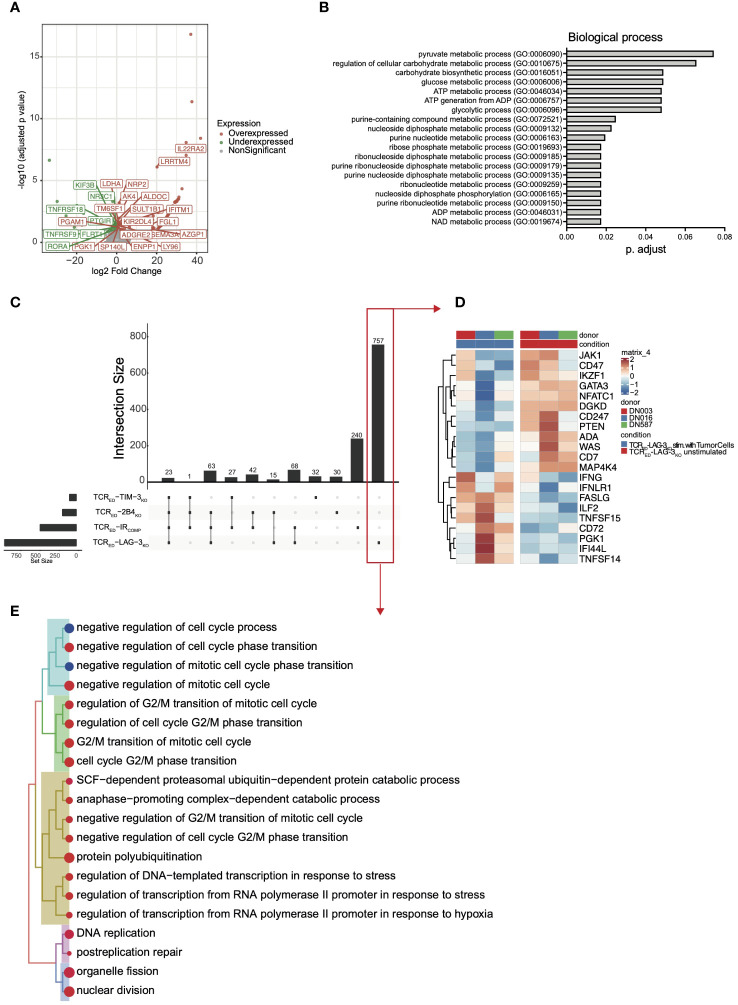
TCR_ED_-IR_KO_ cells display a unique transcriptional profile upon target recognition. Volcano plot showing significant down- (green) and up- (red) regulated genes **(A)** and curated Gene ontology terms (biological process-**(B)** in stimulated TCR_ED_-LAG-3_KO_ vs stimulated TCR_ED_-IR_COMP_ cells. **(C)** UpSet analysis showing shared (connected dots in the lower panel) or unique (not-connected dots in the lower panel) differentially expressed genes in stimulated vs unstimulated TCR_ED_-IR_COMP_, TCR_ED_-LAG-3_KO,_ TCR_ED_-TIM-3_KO,_ TCR_ED_-2B4_KO_ cells. **(D)** Heatmap showing the level of expression of selected genes among the 757 uniquely differentially expressed genes in TCR_ED_-LAG-3_KO_ upon target recognition (red box in panel **(C)**. **(E)** Treeplot of Gene Ontology (Biological Process) terms enriched uniquely in TCR_ED_-LAG-3_KO_ upon target recognition (red box in panel **(C)**). Data are shown for 3 biological replicates for each cellular product, generated from 3 matched healthy donors. MM1.s A2^pos^ESO-1^pos^ multiple myeloma cells were used as antigen source for T cell stimulation.

To shed lights on the mechanisms underlying the functional advantage of TCR_ED_-IR_KO_ cells compared to IR_COMP_ counterparts, we compared the transcriptional profiles of our cellular products upon 24 hrs co-culture with MM1.s A2^pos^ESO-1^pos^ multiple myeloma cells or left unstimulated. First, we analyzed differentially upregulated or downregulated genes in each TCR_ED_-IR_KO_ vs TCR_ED_-IR_COMP_ cells after stimulation with cancer cells. Volcano plot of the differentially expressed genes revealed 183 differentially expressed genes in TCR_ED_-LAG-3_KO_ vs TCR_ED_-IR_COMP_ cells (**Figure 3A**). On the contrary, in the same experimental condition, only 92 and 11 genes were differentially regulated in TCR_ED_-TIM-3_KO_ and TCR_ED_-2B4_KO_ cells respectively, compared with TCR_ED_-IR_COMP_ cells (**Supplementary Figures 5A, B**). Among the 92 differentially expressed genes (DEGs) in TCR_ED_-TIM-3_KO_ T cells, ~1/3 of the DEGs is represented by long non coding RNA (lncRNA) transcripts, suggesting that the lack of IR signaling might reshape lncRNA pattern (44). Gene ontology analysis of the biological process of the 183 DEGs identified in stimulated TCR_ED_-LAG-3_KO_ cells revealed a significant enrichment in glycolytic processes, ADP and NAD metabolism, and nucleotide metabolism, thus suggesting a possible metabolic switch in LAG-3-disrupted antigen specific T cells (**Figure 3B**). To further shed light on the effects of antigen stimulation on our cells, we analyzed the under and overexpressed genes observed in each TCR_ED_, IR_COMP_ and IR_KO_ cellular product upon stimulation with cancer cells. In this analysis, we observed 464 genes differentially expressed in resting vs activated TCR_ED_-IR_COMP_ T cells. Strikingly, this analysis confirmed a significant transcriptional change in TCR_ED_-LAG-3_KO_ T cells, with 926 differentially expressed genes after antigen stimulation. On the contrary, only a few DEGs emerged in TCR_ED_-TIM-3_KO_ T cells and TCR_ED_-2B4_KO_ T cells (**Figure 3C**). Pathway enrichment analysis of the 174 DEGs identified in stimulated TCR_ED_-2B4_KO_ cells, using Gene Ontology-Biological Process as reference, revealed the enrichment of pathways involved in response to pro-inflammatory stimuli, DNA-replication and protein secretion (**Supplementary Figure 5C**). We then focused on genes uniquely up or downregulated in each different cellular product upon antigen exposure.

A total of 757 genes were uniquely differentially expressed in TCR_ED_-LAG-3_KO_ cells (**Figure 3C** red box and **Figure 3D**). Among these, genes associated with T cell activation and effector functions (FASLG, TNFSF15, CD72, PGK1, TNFSF14, IFNG, ILF2) and IFNg response genes (IFI44L, IFNLR1) were significantly upregulated only in TCR_ED_-LAG3_KO_ T cells. Moreover, genes dampening T cell response (IKZF1, CD74 and DGKD (45, 46)) were significantly downregulated exclusively in TCR_ED_-LAG-3_KO_ T cells. Among the downregulated genes, transcription factors (TFs)/TCR-signaling mediators, which are physiologically downregulated upon TCR engagement to preserve homeostatic T cell response (47, 48) (PTEN, GATA3, ADA, WAS, MAP4K4, NFATC1, CD274, JAK1), emerged in TCR_ED_-LAG-3_KO_ T cells (**Figure 3D**). We then performed a pathway enrichment analyses, using Gene Ontology -Biological Process as reference, of the 757 unique DEGs in TCR_ED_-LAG-3_KO_ cells to identify biological processes that were differentially regulated upon antigen recognition by this cellular product. Genes involved in cell cycle, DNA-replication, nucleotide metabolism were significantly upregulated in stimulated TCR_ED_-LAG3_KO_ cells (**Figure 3E**), thus suggesting that, upon target recognition by T cells, LAG-3 disruption might boost the induction of DNA replication. To verify whether TCR triggering induces stronger activation in TCR_ED_-LAG-3_KO_ cells compared to other cellular products, we quantified the phosphorylation of ERK induced by TCR triggering, in our cells (49, 50). According to our hypothesis, we observed a significantly increased phospho-ERK upon activation only in TCR_ED_-LAG-3_KO_ T cells compared to TCR_ED_-IR_COMP_ cells (**Supplementary Figure 5D**). Overall, transcriptomic analysis suggests a different mechanism underlying the effects of LAG-3, TIM-3, and 2B4 genetic loss in T cell products and show that LAG-3 disruption has a major impact on the transcriptional profile of T cells.

### TCR_ED_-IR_KO_ cells outperform TCR_ED_-IR_COMP_ T cells upon chronic antigen stimulation

**Figure 4:**
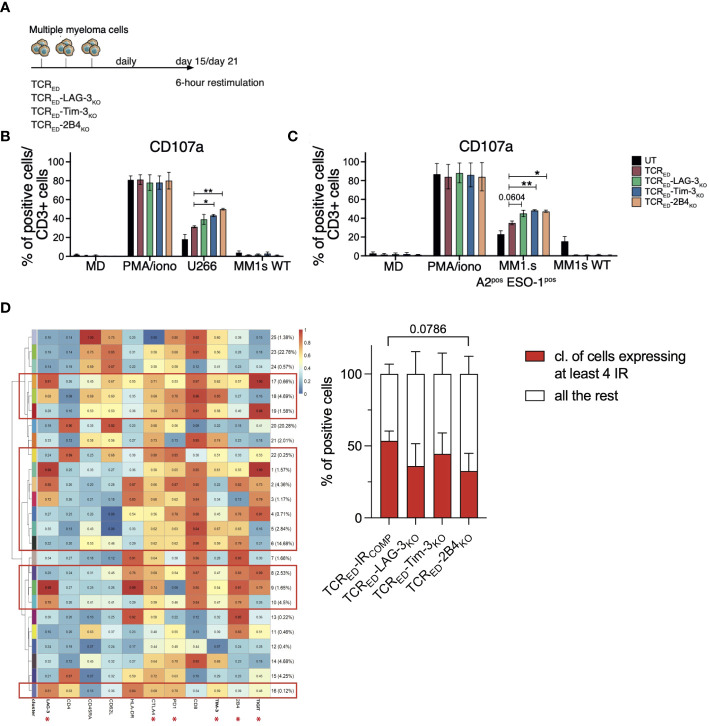
TCR_ED_-IR_KO_ cells outperform TCR_ED_-IR_COMP_ T cells upon chronic antigen stimulation. **(A)** Schematic representation of the T cell exhaustion experiment. **(B, C)** Percentages of degranulating (measured as CD107a^+^) CD3^+^ T cells after 6 hours of restimulation of TCR_ED_-IR_COMP_ (red bars), TCR_ED_-LAG-3_KO_ (green bars), TCR_ED_-TIM-3_KO_ (blue bars), and TCR_ED_-2B4_KO_ (light orange bars) or unmanipulated (UT, black bars) T cells challenged for 15-21 days with U266 or MM1.s A2^pos^ESO-1^pos^ tumor cells. Only medium, PMA/Ionomycin and HLA-A2^neg^NY-ESO-1^neg^ MM1.s wt cells were used as controls (N=3). **(D)** Meta-cluster composition of TCR_ED_-IR_COMP_ and TCR_ED_-IR_KO_ T cells phenotyped after 18 days of daily stimulation with MM1.s A2^pos^ESO-1^pos^ cells (left). The red squares indicate clusters displaying the co-expression of at least 4 IRs among the ones indicated with an asterisk. On the right, the percentages of TCR_ED_-IR_COMP_ and TCR_ED_-IR_KO_ T cells co-expressing ≥ 4 IRs at the end of chronic antigen stimulation is shown. Data are shown as mean ± SEM of 3 biological replicates for each cellular product.*: p value <0.05; **: p-value < 0.01; ***: p-value < 0.001; ****: p-value < 0.0001.

To model T cell exhaustion and verify whether the lack of TIM-3-, LAG-3- or 2B4 overcome the loss of function of tumor-specific T cells, we adapted a chronic stimulation protocol (51–53) and daily stimulated TCR_ED_-IR_COMP_ and TCR_ED_-IR_KO_ T cells with U266 or MM1.s A2^pos^ESO-1^pos^ cancer cells, and we assessed the anti-tumor response 2 to 3 weeks later (**Figure 4A**). In these stressed conditions, TCR_ED_-TIM-3_KO_ and TCR_ED_-2B4_KO_ lymphocytes displayed a higher degranulation capacity than TCR_ED_-IR_COMP_ counterparts, when challenged with U266 (**Figure 4B**) or MM1.s A2^pos^ESO-1^pos^ cell lines (**Figure 4C**), indicating that the lack of TIM-3 and 2B4 in tumor-specific T cells sustains prolonged effector functions and overcomes exhaustion upon chronic antigen exposure. Despite ligands expression on U266 and MM1.s tumor cells, LAG-3 disruption didn’t appear to impact on TCR_ED_ function in these assays. Of notice, no significant differences were observed in IL-2, perforin, sFASL nor TNFα secretion in the different T cell products (**Supplementary Figure 6**).

To further investigate how the lack of IR-mediated signaling may affect chronically stimulated tumor-specific T cells, we phenotyped TCR_ED_-IR_KO_ cells and IR competent counterparts at the end of chronic stimulation with MM1.s A2^pos^ESO-1^pos^. Unsupervised analysis was used to identify the clusters of events based on the co-expression of IRs, and, in particular, the percentage of cells expressing more than 4 IRs among LAG-3, CTLA4, PD-1, TIM-3, 2B4, and TIGIT was calculated on each T cell product. TCR_ED_-IR_KO_ cells display a reduced, though not significant, number of IRs on the cell surface, compared to TCR_ED_-IR_COMP_ counterparts (**Figure 4D**). These observations suggest that the disruption of IRs might prevent the upregulation of additional inhibitory receptors upon chronic antigenic stimulation and, overall, indicate that the disruption of different IRs affects distinct T cell effector functions.

### TCR_ED_-IR_KO_ cells outperform TCR_ED_-IR_COMP_ T cells against multiple myeloma *in vivo*

To verify *in vivo* the advantages of the lack of IRs, we set up a model in which mice with high tumor burden were treated with a limited doses of tumor-specific T cells. In this setting, cancer cells represent a continuous source of T cell activation that could potentially trigger T cell exhaustion, similarly to what occurs in cancer patients. Considering the high expression of LAG-3 ligands measured in U266 cells (**Supplementary Figure 3**) with this model we initially compared TCR_ED_-IR_COMP_ vs TCR_ED_-LAG3_KO_ T cells. 10x10^6^ luciferase^pos^ U266 cells, expressing high levels of LAG-3 ligands, were injected in sub-lethally irradiated NSG mice. When high-tumor burden was detected by total body bioluminescence imaging (BLI), mice were treated with two different doses (10^6^ or 5x10^6^) of either TCR_ED_-LAG-3_KO_ or TCR_ED_-IR_COMP_ effector cells. Control mice were left untreated (nil) or treated with untransduced cells (UT, 5x10^6^ dose) (**Supplementary Figure 7A**). The lowest dose of lymphocytes was not sufficient for controlling tumor growth independently from the infused cellular product. Instead, 5x10^6^ TCR_ED_-IR_COMP_ T cells induced a partial tumor control, while 5x10^6^ TCR_ED_-LAG-3_KO_ T cells completely eradicated the tumor (**Supplementary Figure 7B**). The frequencies of circulating human lymphocytes increased with the dose infused (**Supplementary Figure 7C**). A similar growth kinetic was observed with both TCR_ED_ cellular products, while untransduced cells, although ineffective in controlling the disease, further expanded as observed in xeno-GvHD models (54, 55). Interestingly, a significant correlation was detected between the tumor burden and the expansion of TCR_ED_-LAG-3_KO_ T cells, but not with TCR_ED_-IR_COMP_ T cells, suggesting a dose-dependent tumor killing in the absence of LAG-3 (**Supplementary Figure 7D**).

Although not sufficient for tumor eradication, TCR_ED_-LAG-3_KO_ circulating in mice treated with the lowest T cell dose maintained a higher activation profile than TCR_ED_-IR_COMP_ cells (**Supplementary Figure 7E**), indicating a preserved ability to respond upon prolonged antigen exposure. Accordingly, at sacrifice, 3 weeks after T-cell infusion, a higher proportion of TCR_ED_-IR_COMP_ cells infiltrating the bone marrow co-expressed multiple IRs compared to TCR_ED_-LAG-3_KO_ cells (**Supplementary Figure 7F**), indicating that LAG-3 disruption in T cells redirected against NY-ESO-1 curtails the upregulation of other inhibitory receptors, even in stressed conditions of tumor outgrowth.

Interestingly, although U266 cells expressed lower levels of TIM3 and 2B4 ligands than MM1s cells *in vitro* (**Supplementary Figure 3**), when we analyzed the profile of U266 cells harvested from mice, we observed an upregulation of CD48, HLA-DR and CEACAM (**Supplementary Figures 8A–D**) and we measured detectable levels of HMGB1 and Galectin-9 in the serum (**Supplementary Figure 8E**). Thus, we reasoned that U266 could represent an appropriate model to compare all our TCR_ED_-IR_KO_ cellular products.

**Figure 5:**
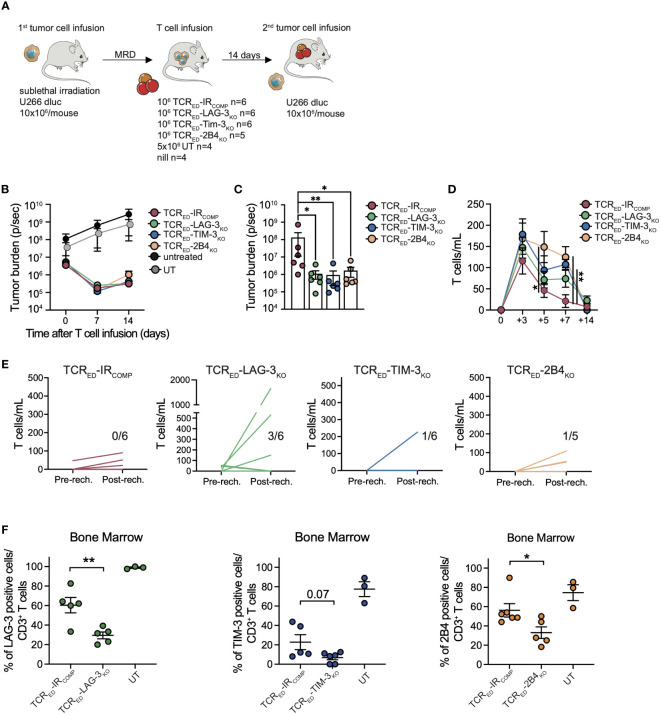
TCR_ED_-IR_KO_ cells outperform TCR_ED_-IR_COMP_ T cells against multiple myeloma *in vivo.* **(A)** Schematic representation of *in vivo* rechallenge experiment with U266 multiple myeloma cells. Tumor burden evaluated by total body bioluminescence (BLI) during **(B)** the first anti-tumor response and **(C)** after re-challenge with U266, in mice treated with low dose of TCR_ED_-IR_COMP_ (red lines), TCR_ED_-LAG-3_KO_ (green lines), TCR_ED_-TIM-3_KO_ (blue lines), TCR_ED_-2B4_KO_ (orange lines) T cells. In panel B tumor bearing mice left untreated (black) or injected with untransduced lymphocytes (grey) are shown. Absolute quantification of circulating CD3^+^ cells before **(D)** and after **(E)** tumor rechallenge. In panel **E** each line represents a single mouse and the number in each graph indicates the number of mice with human CD3 total cells/mL higher than 100. **(F)** Frequency of human T cells expressing TIM-3 (blue), LAG-3 (green) and 2B4 (orange) in the bone marrow of treated mice at sacrifice. Data are shown as mean ± SEM. N. of treated and analyzed animals per group are shown in **(A)**. *: p value <0.05; **: p value < 0.01; ***: p value < 0.001.

To verify whether the lack of LAG-3, TIM-3, or 2B4-mediated signaling in TCR_ED_ T cells prevents functional exhaustion and promotes immunological memory, the anti-tumor responses of TCR_ED_-IR_COMP_, TCR_ED_-LAG-3_KO_, TCR_ED_-TIM-3_KO_ and TCR_ED_-2B4_KO_ cells were compared in a tumor rechallenge model. Sub-lethally irradiated NSG mice were infused with luciferase^pos^ U266 cells and treated with low doses (1x10^6^/mouse) of engineered (TCR_ED_-IR_COMP_, TCR_ED_-LAG-3_KO_, TCR_ED_-TIM-3_KO_, TCR_ED_ *-*2B4_KO_ T cells) or untransduced cells in a minimal residual disease setting (total flux <= 10^6^) (**Figure 5A**). As shown in **Figure 5B**, **Supplementary Figure 9A**, all edited cellular products completely eradicated the tumors in 2 weeks, while animals receiving untransduced lymphocytes displayed a massive tumor growth. We then rechallenged all mice treated with edited lymphocytes with 10x10^6^ U266 cells. Impressively, all TCR_ED_-IR_KO_ cells, but not TCR_ED_-IR_COMP_ cells, were able to completely eradicate tumor cells (**Figure 5C**, **Supplementary Figure 9A**), thus demonstrating enhanced T cell fitness *in vivo*. Despite a similar kinetic of T cell expansion observed with all engineered effectors upon T-cell infusion (**Figure 5D**), after tumor rechallenge, TCR_ED-_LAG-3_KO_ T cells displayed the highest and most consistent expansion profile (**Figure 5E**), in accordance with their transcriptional profile. Interestingly, at sacrifice, a significantly higher proportion of activated CD3^+^ cells were present in the spleens of all TCR_ED_-IR_KO_ treated mice compared to TCR_ED_-IR_COMP_ treated mice (**Supplementary Figure 9B**). In addition, human T cells displayed a similar differentiation phenotype, dominated by effector cells but with a measurable proportion of early memory (T_SCM_ and T_CM_) lymphocytes (**Supplementary Figure 9C**). Of interest, the majority of BM-T cells remained negative for each genetically disrupted IR (**Figure 5F**), indicating the absence of a counterselection of IR edited cellular products in this experimental setting.

Overall, our data indicate that LAG-3, TIM-3, or 2B4 disruption not only prevents the upregulation of additional inhibitory molecules and enhances the cytotoxic activity upon chronic stimulations, but also supports a prolonged anti-tumor activity.

## Discussion

The long-term efficacy of adoptive T cell therapy with TCR gene-edited or CAR-T cells is still limited by T cell exhaustion and by limited persistence of infused cellular products. Here, we aimed at overcoming these hurdles by developing innovative cellular products redirected against a tumor-antigen, endowed with long-term persistence potential and resistant to inhibitory pathways.

In cancer patients, tumor-specific T cells are chronically stimulated by tumor antigens in an immunosuppressive tumor microenvironment (TME) and acquire an exhausted phenotype. Loss of effector functions in exhausted cells is triggered by different pathways downstream to multiple inhibitory receptors that are expressed on the cell surface, including TIM-3, LAG-3 and 2B4. To foster immune escape, cancer cells upregulate IR ligands and exploit these inhibitory pathways to dampen T cell responses. Checkpoint inhibitors revert T cell exhaustion by restoring T cell proliferation and pro-inflammatory cytokine production, thus rescuing effective anti-tumor responses. Although the use of ICBs in clinical studies induces prolonged tumor-free survival in a fraction of treated patients, their use is often limited by the occurrence of severe autoimmune related adverse events. This secondary unwanted reaction is mainly due to the lack of specificity of ICB, which may unleash autoreactive T cells. To overcome these limitations, we simultaneously disrupted the endogenous TCR α chain in combination with TIM-3, LAG-3 or 2B4 and we generated TCR_ED_-IR_KO_ cells by transduction with a lentiviral vector encoding for a HLA-A2 restricted NY-ESO-1 specific TCR (56). With our optimized biotechnological tools, we were able to obtain highly efficient gene disruption and transduction.

Although chromosomal translocations derived from bi- or tri-genic modifications have been reported in clinical trials with genetically engineered cells (23, 57), their frequency was comparable to that observed in healthy individuals (58–60) and no secondary malignant transformation associated to translocations was reported in these trials (23, 57). While long-term follow up of treated patients will provide further insights into the safety profile of CRISPR/Cas9 nucleases, other alternative gene editing tools that do not require DNA double strand break induction, such as the recently developed base editing and prime editing platforms, could be used to further reduce the risk of translocations when editing multiple genes. In our study, we employed a high-fidelity version of Cas9, and expect to detect low numbers of off-targets in our cellular products. Although specificity will need to be evaluated before clinical implementation, no differences in the expansion capacity were detected *in vitro* or *in vivo* among different engineered T cells, indicating that no significant perturbation of T cell fitness occurs upon multiple gene disruptions.

Durable clinical responses strongly rely on the persistence of adoptively transferred cells. T_SCM_ and T_CM_ cells are endowed with self-renewal, high-proliferative potential and superior long-term persistence, thus representing ideal subsets for ACT (61–65). Here, we combined genetic engineering with a stimulation and culture protocol that supports the expansion of T_SCM_/T_CM_ cells (34), and we observed that multiple gene disruptions by CRISPR/Cas9 in combination with TCR gene editing is feasible in T_SCM_ cells without altering their phenotype, expansion, or proliferative capacities. Notably, our results indicate that TCR_ED_-IR_KO_ cells retain an early differentiated phenotype, thus suggesting that TIM-3, LAG-3 and 2B4 disruptions do not impact the memory differentiation of edited cells. Accordingly, a measurable proportion of T_SCM_ cells could be also quantified in the bone marrow of mice treated with our engineered cellular products.

The disruption of inhibitory receptors in tumor-specific T cells has been recently proposed (23–31, 66). In these studies, the authors abrogated the expression of PD-1, CTLA-4, LAG-3, A2AR or CD39 encoding genes in engineered T cells. While a slight increase in survival was shown in mice treated with IR-disrupted cells, conclusive results on the impact of each IR disruption on tumor-redirected T cells in tumor control upon chronic stimulation are lacking. Furthermore, a comparative analysis of cellular products devoid of single IR has not been reported. In a clinical trial with PD-1 disrupted cells edited for T cell specificity, the frequency of cells with edits in the *PDCD1* locus decreased from 25% to 5% of the cells expressing the transgenic TCR at 4 months after infusion, consistently with mouse studies of chronic infection where PD-1 deficient T cells proved less able to establish memory (23). However, recent reports challenged this hypothesis and demonstrated in a murine model long-term persistence and function of murine adoptively transferred antigen-specific T cells with genetically ablated PD-1 expression (67).

The TCR_ED_-IR_KO_ cells developed in the present study provide a unique platform to compare the impact of different IRs and proliferative capacity of human T-cell based products. A single antigen specificity allowed the assessment of antitumor activity without the confounding factors of unspecific or allo-reactivities or xeno-reactivity, and also permitted to shed light on TIM-3-, LAG-3-, or 2B4-mediated exhaustion mechanisms in antigen-specific human T cells. Furthermore, the evaluation of cytotoxicity in short-term assays showed that TCR_ED_-TIM-3_KO_, TCR_ED_-LAG-3_KO,_ and TCR_ED_-2B4_KO_ cells display comparable killing activity to TCR_ED_-IR_COMP_ cells. These results indicate that IR-disrupted T cells retain their killing capacity, thus proving that TIM-3, LAG-3, or 2B4 are not required on T cells for effective effector functions.

Although originally interpreted as a homogeneous state of T cell dysfunction, T cell exhaustion appears today as a progressive developmental path, characterized by different cell states, with heterogeneous functional phenotypic profiles (68, 69). In line with this notion, in our study, effects of IR_KO_ in our cells were heterogenous, and amplified upon increase in the complexity of the experimental conditions. In short term culture, TCR_ED_-IR_KO_ cells were similar to TCR_ED_-IR_COMP_ cells in killing efficiency and degranulation ability, but with increased capacity to secrete pro-inflammatory cytokines. Upon chronic antigen stimulation, TCR_ED_-TIM-3_KO_ and TCR_ED_-2B4_KO_ lymphocytes showed a higher degranulation capacity than TCR_ED_-IR_COMP_ counterparts. Finally, upon *in vivo* tumor rechallenge, all TCR_ED_-IR_KO_ proved superior to TCR_ED_-IR_COMP_ counterparts in killing cancer cells.

Although IRs have been extensively investigated in infectious and cancer models, the role of each individual inhibitory receptor has not been compared in tumor-specific cellular products. This side-by-side comparison would be pivotal in the development of tailored and effective cellular products for cancer patients. Here, although a similar positive effect was detected in all TCR_ED_-IR_KO_ cells, upon chronic *in vitro* antigen stimulation and *in vivo*, data indicate that the disruption of TIM-3, LAG-3, or 2B4 might affect different T cell effector functions. Gene expression analysis uncovered enrichment of genes involved in proliferation and cell cycle upon LAG-3 disruption, and of genes associated with effector response in 2B4-disrupted T cells. Interestingly, the few DEGs identified in TCR_ED_-TIM-3_KO_ T cells, were highly enriched for long non coding RNA (lncRNA) transcripts. This observation is in line with a recent report showing specific lncRNA regulating TIM-3 signaling in TILs (44) and supports the notion that inhibitory receptors dampen T cell responses through distinct mechanisms.

Chronic stimulation with low Ag burden has been reported to model T cell exhaustion *in vitro* (51–53). The disruption of TIM-3 and 2B4 increases the degranulation capacity of NY-ESO-1 specific T cells upon chronic stimulation with multiple myeloma cells. Since exhausted T cells often co-express several IRs, multiple IR gene disruption might be beneficial. Based on our *in vitro* and *in vivo* observations, and based on the results of trascriptomic data, we propose LAG-3/TIM-3 and LAG-3/2B4 kock-out combinations, to be prioritized based on the frequency and relevance of TIM-3 and 2B4 in the selected diseases.

In a high tumor burden setting, designed to foster T cell exhaustion, we observed that TCR_ED_-LAG-3_KO_ cells infiltrating the bone marrow in conditions of antigen persistence display a reduced induction of compensatory inhibitory molecules (CTLA-4, PD-1 and TIM-3), thus further supporting our *in vitro* findings. In the same model, high doses of TCR_ED_-LAG-3_KO_ cells mediated a significantly higher anti-tumor activity than TCR_ED_-IR_COMP_ cells.

The final aim of our study was to reveal whether IR disruptions in our cellular products provide resistance to T-cell exhaustion and promote functional immunological memory. To this aim, the anti-tumor responses of TCR_ED_-IR_COMP_, TCR_ED_-LAG-3_KO_, TCR_ED_-TIM-3_KO_ and TCR_ED_-2B4_KO_ cells were compared in a tumor rechallenge model. Upon tumor control in mice, we challenged T cells *in vivo* with a second infusion of tumor cells. Only TCR_ED_-IR_KO_ cells were able to completely eradicate the second tumor engraftment, indicating their ability to resist functional exhaustion. The enhanced fitness of TCR_ED_-IR_KO_ cells was also reflected by the higher proportion of activated IR_KO_ tumor-specific T cells infiltrating the bone marrow compared to TCR_ED_-IR_COMP_ cells, at sacrifice. Interestingly, in accordance with the transcriptional findings, TCR_ED-_LAG-3_KO_ T cells displayed the highest and most consistent expansion profile, after tumor rechallenge, further confirming a unique mechanism of action for LAG3_KO_ cellular products.

Limitations of our study include the lack of stable and reliable surface markers to quantify by flow cytometry IR-disrupted cells and the fact that immunodeficient mice models cannot recapitulate the entire tumor microenvironment. Additional information may be gained by future investigation of IR_KO_ in additional tumor models, additional immunodeficient models and in immunocompetent mice. Our study provides a novel and unique platform to test the impact of single IR disruptions on antigen-specific cellular products and to shed lights on their mechanisms of action in clinically relevant models. In future developments, the fine-tuning of multiple IR disruptions tailored for specific tumor types and able to foster active proliferation in long-living memory cells will ultimately generate better cellular products for disease-specific adoptive T cell therapy approaches.

## Data availability statement

The data presented in the study are deposited in the Gene Expression Omnibus (GEO) repository, accession number GSE256035.

## Ethics statement

The studies involving humans were approved by San Raffaele Scientific Institutional Ethical Committee. The studies were conducted in accordance with the local legislation and institutional requirements. The human samples used in this study were acquired from primarily isolated as part of your previous study for which ethical approval was obtained. Written informed consent for participation was not required from the participants or the participants’ legal guardians/next of kin in accordance with the national legislation and institutional requirements. The animal study was approved by Institutional Animal Care and Use Committee (IACUC). The study was conducted in accordance with the local legislation and institutional requirements.

## Author contributions

BCC: Conceptualization, Formal analysis, Funding acquisition, Investigation, Validation, Visualization, Writing – original draft. ZIM: Investigation, Methodology, Validation, Visualization, Resources, Writing – review & editing. AU: Investigation, Methodology, Validation, Visualization, Resources, Writing – review & editing. BC: Investigation, Resources, Methodology, Writing – review & editing. IM: Data curation, Formal Analysis, Writing – review & editing. ES: Data curation, Formal Analysis, Writing – review & editing. LP: Investigation, Writing – review & editing. AS: Investigation, Writing – review & editing. AP: Conceptualization, Formal Analysis, Investigation Visualization, Writing – review & editing. VV: Conceptualization, Formal Analysis, Investigation, Writing – review & editing. AI: Conceptualization, Formal Analysis, Investigation, Writing – review & editing. FM: Formal Analysis, Software, Writing – review & editing. DA: Formal Analysis, Software, Writing – review & editing. LV: Conceptualization, Writing – review & editing. RDM: Conceptualization, Writing – review & editing. FC: Conceptualization, Writing – review & editing. LN: Conceptualization, Writing – review & editing. PG: Conceptualization, Writing – review & editing. ER: Conceptualization, Formal Analysis, Funding acquisition, Project administration, Supervision, Visualization, Writing – review & editing. CB: Conceptualization, Formal Analysis, Funding acquisition, Project administration, Supervision, Visualization, Writing – review & editing.
