# Supplementary material for: TIM-3, LAG-3, or 2B4 gene disruptions increase the anti-tumor response of engineered T cells
Source: Front Immunol. 2024 Feb 29;15:1315283. doi: 10.3389/fimmu.2024.1315283 (PMC10953820; doi:10.3389/fimmu.2024.1315283)
Supplement: Supplementary file 1 [file DataSheet_1.pdf]

*Supplementary Material*

# TIM-3, LAG-3, or 2B4 gene disruptions increase the anti-tumor response of engineered T cells

**Beatrice Claudia Cianciotti, Zulma Irene Magnani†, Alessia Ugolini†, Barbara Camisa, Ivan Merelli, Valentina Vavassori, Alessia Potenza, Antonio Imparato, Francesco Manfredi, Danilo Abbati, Laura Perani, Antonello Spinelli, Eric Shifrut6 Fabio Ciceri, Luca Vago, Raffaella Di Micco, Luigi Naldini, Pietro Genovese, Eliana Ruggiero‡* and Chiara Bonini*‡.**

**†these authors contributed equally to this work; ‡ these authors contributed equally to this**

*** Correspondence:** bonini.chiara@hsr.it, ruggiero.eliana@hsr.it

# 1 Supplementary tables

**Table 1: List monoclonal antibodies**

| Specificity | Antibody | Color | Clone | Catalogue | Source | Experiment |
|---|---|---|---|---|---|---|
| anti Human | 2B4 (CD244) | PE-dazzle | C1,7 | 329522 | BioLegend | C/E/I |
| anti Human | 7AAD | | | 420404 | BioLegend | E/L |
| anti Human | Annexin V | Alexa fluor 647 | | 640912 | BioLegend | L |
| anti Human | CD107a (LAMP-1) | PE eFluor610 | eBioH4A3 | 61-1079-42 | Invitrogen | C/E |
| anti Human | CD138 | PE | DL-101 | 352306 | BioLegend | I |
| anti Human | CD138 | PE/Cy5 | B-A38 | A54191 | Beckman Coulter | L |
| anti Human | CD160 | PE/Cy7 | BY55 | 341212 | BioLegend | E |
| anti Human | CD25 | APC/Cy7 | BC96 | 302614 | BioLegend | C/E |
| anti Human | CD3 | BV510 | OKT3 | 317332 | BioLegend | C/E/I |
| anti Human | CD3 | FITC | SK7 | 11-0036-42 | Invitrogen | C/E |
| anti Human | CD3 | PE/Cy7 | OKT3 | 25-0037-42 | Invitrogen | C/E |
| anti Human | CD3 | BUV661 | SK7 | 741692 | BD Biosciences | C/E |
| anti Human | CD3 | PerCP | UCHT1 | MA1-10181 | Invitrogen | P |
| anti Human | CD38 | PE/Cy7 | HB-7 | 356608 | BioLegend | I/L |
| anti Human | CD39 | BUV737 | TU66 | 58781 | BD Biosciences | E |
| anti Human | CD4 | APC/Cy7 | OKT4 | 317418 | BioLegend | C/E |
| anti Human | CD4 | APC | OKT4 | 17-0048-42 | Invitrogen | C/E |
| anti Human | CD4 | PE/Cy7 | OKT4 | 25-0048-42 | Invitrogen | E |

| | | | | | | |
|---|---|---|---|---|---|---|
| anti Human | CD40L (CD154) | PE | TRAP1 | 555700 | BD Biosciences | C/E |
| anti Human | CD45RA | BUV496 | H100 | 750258 | BD Biosciences | C/E |
| anti Human | CD45RA | PE | H100 | 12-0458-42 | Invitrogen | E |
| anti Human | CD48 | APC Vio770 | REA426 | 130-106-470 | Miltenyi | I/L |
| anti Human | CD62L | BUV737 | DREG-56 | 741843 | BD Biosciences | C/E |
| anti Human | CD62L | Pacific Blue | DREG-56 | 304826 | BioLegend | E |
| anti Human | CEACAM (CD66a/c/e) | PE | ASL-32 | 342304 | BioLegend | L |
| anti Human | CD69 | APC | FN50 | 310910 | BioLegend | C/E |
| anti Human | CD8 | BV785 | SK1 | 344740 | BioLegend | C/E |
| anti Human | CD8 | PerCP | SK1 | 345774 | BD | C/E |
| anti Human | CD8 | APC/Cy7 | SK1 | 344714 | BioLegend | C/E |
| anti Human | CD8 | BUV395 | RPA-T8 | 9240259 | BD Biosciences | E |
| anti Human | CD80 | PE/Cy7 | 2D10 | 305218 | BioLegend | I |
| anti Human | CD95 (FAS) | FITC | DX2 | 305606 | BioLegend | E |
| anti Human | CTLA-4 (CD152) | BV605 | BNI3 | 369610 | BioLegend | C/E/I |
| anti Human | DAPI | | | D9542 | Sigma-aldrich | C/E/I |
| anti Human | ERK1/2 (pT202/pY204) | | 20A | 562981 | BD Biosciences | P |
| anti Human | Galectin-9 | APC | 9M1-3 | 348908 | BioLegend | L |
| anti Human | hCD45 | APC/Cy7 | H130 | 304014 | BioLegend | I |
| anti Human | HLA-A2 | PE | BB7.2 | 12-9876-42 | Invitrogen | I |
| anti Human | HLA-A2 | APC | BB7.2 | 343308 | BioLegend | L |
| anti Human | HLA-DR | BV480 | G46-6 | 566113 | BD Biosciences | C/E/I |
| anti Human | HLA-DR | PE | L243 | 307606 | BioLegend | C/E |
| anti Human | HLA-DR | APC/Cy7 | L243 | 307618 | BioLegend | E |

| | | | | | | |
|---|---|---|---|---|---|---|
| anti Human | IFNg | APC/Cy7 | 4S.B3 | 502530 | BioLegend | C/E |
| anti Human | IL2 | APC | MQ1-17H12 | 500310 | BioLegend | C/E |
| anti Human | Ki-67 | Alexa fluor 488 | Ki-67 | 350508 | BioLegend | C/E |
| anti Human | KLRG1 (MAFA) | BV785 | 2F1/KLRG1 | 138429 | BioLegend | E |
| anti Human | LAG3 (CD223) | APC | REA351 | 130-119-567 | Miltenyi | C/E/I |
| anti Mouse | mCD45 | PerCP | 30-F11 | 103130 | BioLegend | I |
| anti Human | NGFR (CD271) | APC | ME20.4 | 17-9400-42 | Invitrogen | L |
| anti Human | PD-1 (CD279) | BV650 | EH12-2H7 | 329950 | BioLegend | C/E/I |
| anti Human | PD-1 (CD279) | PE/Cy7 | EH12.2H7 | 329918 | BioLegend | C/E |
| anti Human | PD-1 (CD279) | APC | EH12.2H7 | 329908 | BioLegend | E |
| anti Human | PD-1 (CD279) | BV421 | EH12-2H7 | 329920 | BioLegend | C/E |
| anti Human | TIGIT | PE/Cy7 | MBSA43 | 25-9500-42 | Invitrogen | C/E |
| anti Human | TIGIT | BB700 | 741182 | 747846 | BD Biosciences | E |
| anti Human | TIM3 (CD3663) | PE | REA635 | 130-119-785 | Miltenyi | C/E/I |
| anti Human | TNFa | PE/Cy7 | MAb11 | 502930 | BioLegend | C/E |
| anti Human | Vβ13.1 | FITC | | IM1554 | Beckman Coulter | E |
| | Zombie aqua | BV510 | | 423102 | BioLegend | L |
| | Zombie violet | Pacific blue | | 423114 | BioLegend | C/E/I |

**Legend:** C= chronic; E= editing; I= in vivo; L= cell lines; P= phosphoFlow

## 2 Supplementary Figures

# Supplementary Figure 1

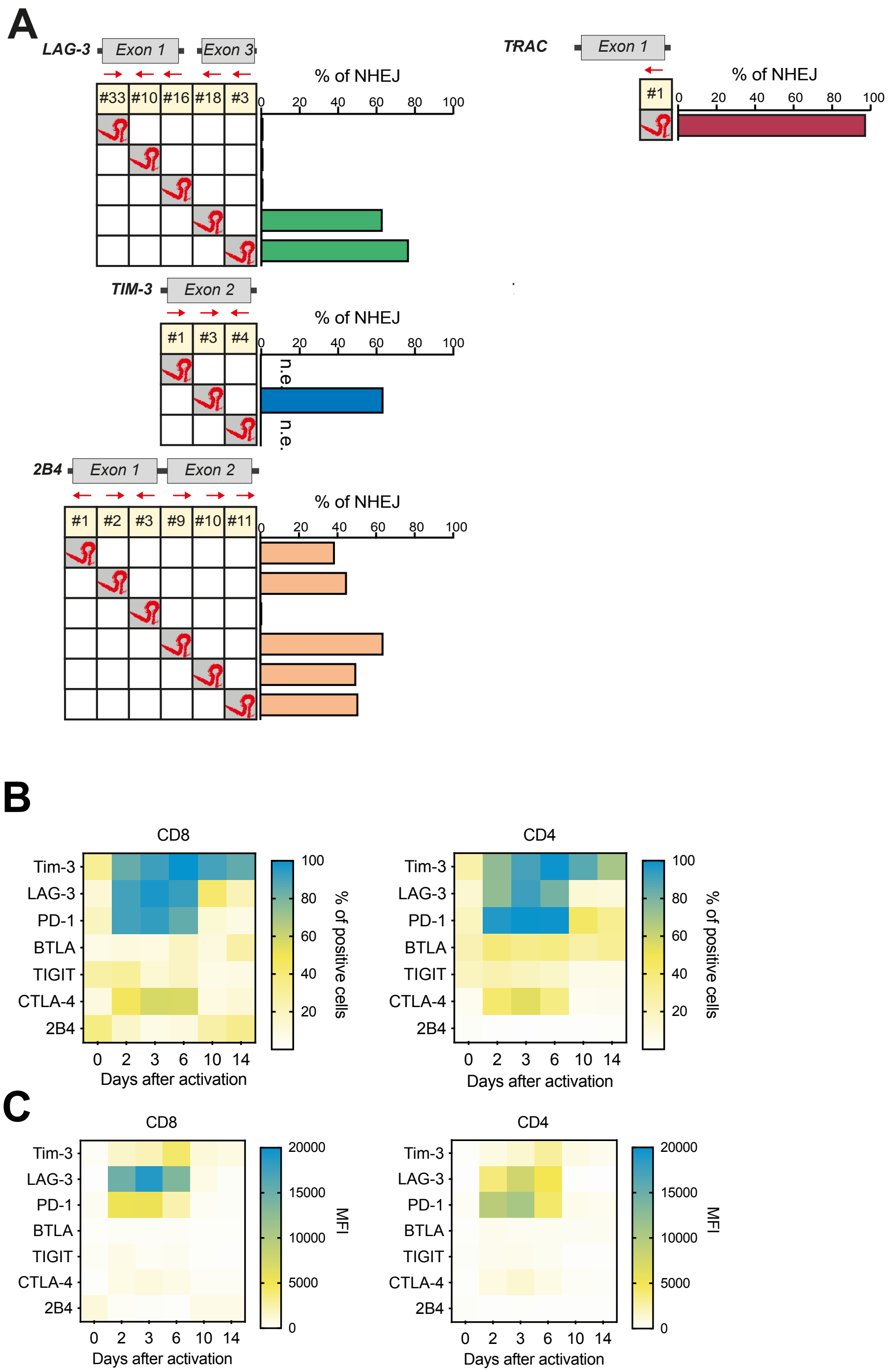

**Supplementary Figure 1. Design and screening of multiple gRNAs targeting the endogenous TCR or inhibitory genes.**

(**A**) Screening of gRNAs targeting the coding regions of *LAG-3*, *TIM-3*, *2B4*, and *TRAC* genes. For each gRNAs, the frequency of NHEJ events is shown. Each red arrow indicates a single gRNA and the direction of the arrows indicate the genomic orientation of the gRNA sequences. Heatmap showing the frequencies (**B**) and the mean fluorescence intensity-MFI (**C**) of $IR^+$ $CD8^+$ (left) and $CD4^+$ (right) T cells in resting conditions (day 0) and in the first 14 days after polyclonal stimulation. The percentage of positive cells and the MFI is indicated by a color gradient ranging from white (0%) to blue (100%), as indicated in the right panel. Data from a single healthy donor were used for gRNA screening (A). In panel B and C, data from 3 different biological replicates are shown (N=3).

## Supplementary Figure 2

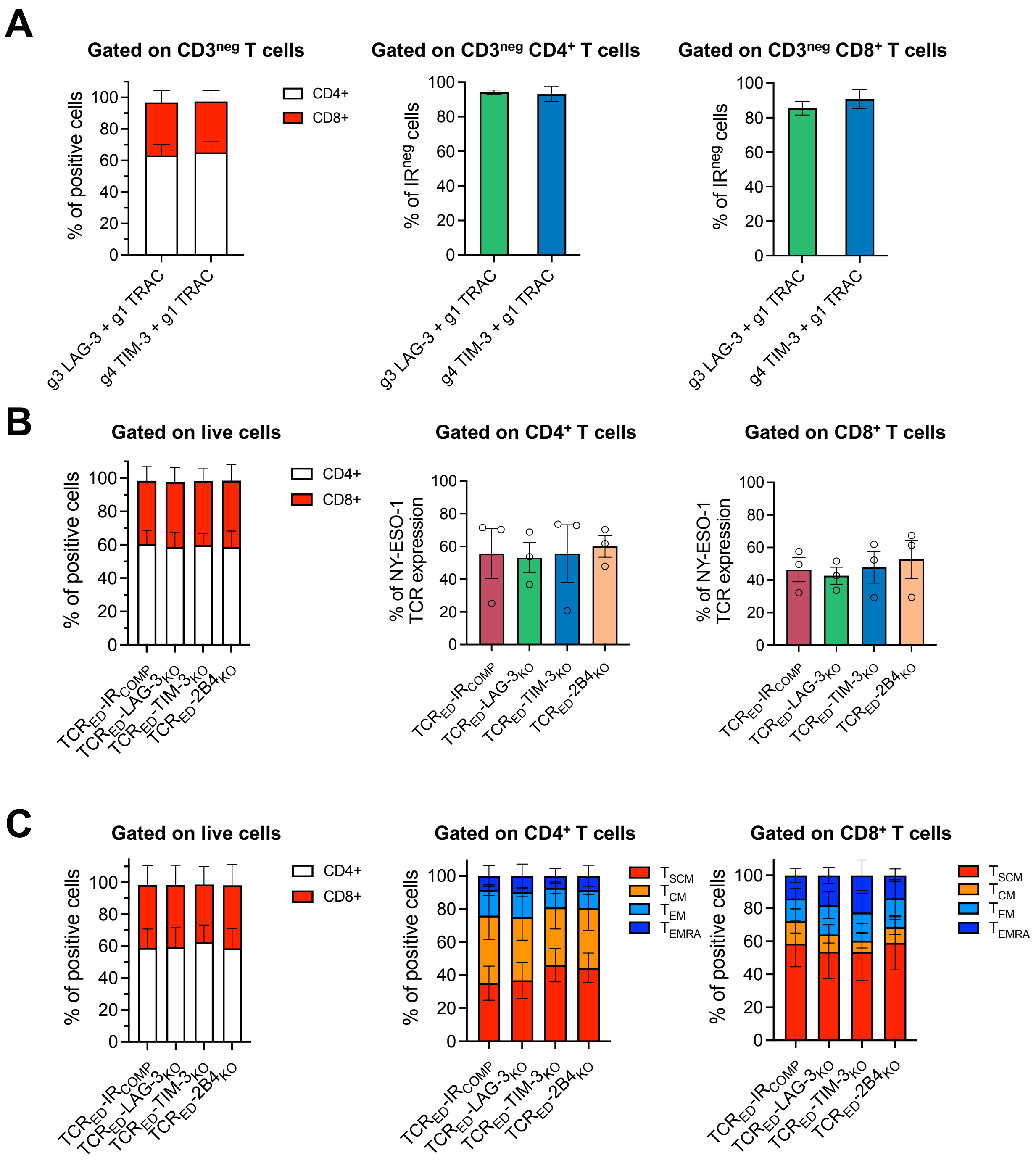

**D**

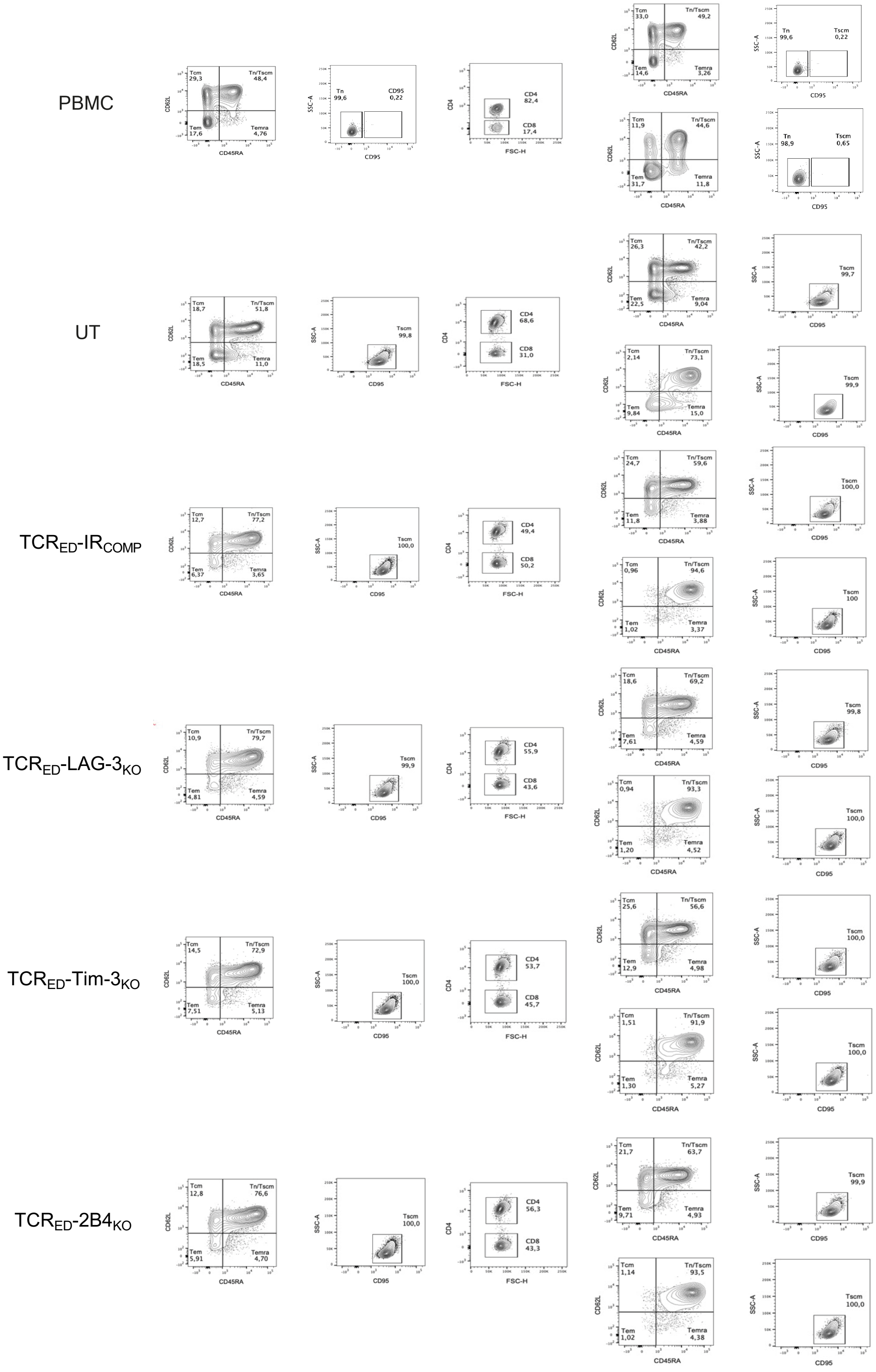

**Supplementary Figure 2. Gene modifications and memory compositions of CD4$^+$ and CD8$^+$ TCR$_{ED}$-IR$_{KO}$ T cells.**

(**A**) Bar graphs showing the frequencies of CD4$^+$ and CD8$^+$ T cells (left panel), the frequencies of LAG3$^{neg}$ (green bars) and TIM-3$^{neg}$ (blue bars) CD4$^+$ and CD8$^+$ T cells (central and right panels, respectively) in CD3$^{neg}$ T cells 5 days after CRISPR/Cas9 delivery (day 7 of the manufacturing protocol) in the indicated cellular products (N=3). (**B**) Bar graphs showing the frequencies of CD4$^+$ and CD8$^+$ T cells (left panel) and the frequencies of CD4$^+$ and CD8$^+$ cells (central and right panels, respectively) expressing the NY-ESO-1-specific TCR at day 10 of the manufacturing protocol in the indicated cellular products (N=3). (**C**) Bar graphs showing the frequency of CD4$^+$ and CD8$^+$ T cells (N=3) and the memory phenotype of CD4$^+$ and CD8$^+$ T cells (N=4) (central and right panel, respectively) at the end of the manufacturing protocol (day 18) in all tested cellular products. Data are shown as mean ± SEM from 3-4 different biological replicates. (**D**) Representative dot plots depicting the frequencies of effector and memory CD3$^+$, CD4$^+$, CD8$^+$ T cell subsets in TCR$_{ED}$-IR$_{COMP}$ and TCR$_{ED}$-IR$_{KO}$ cells.

# Supplementary Figure 3

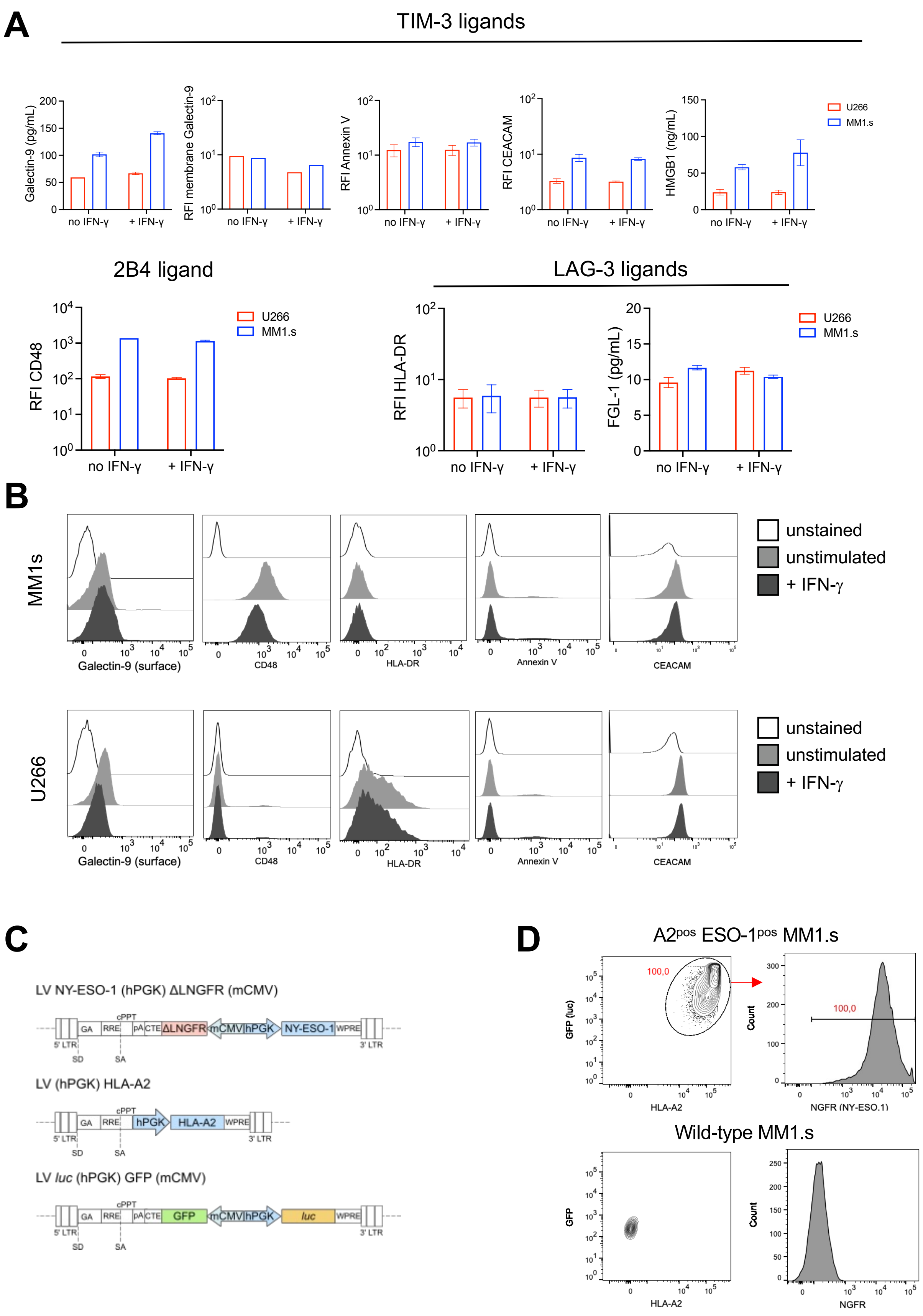

**Supplementary Figure 3. Generation and characterization of HLA-A2$^{POS}$ NY-ESO-1$^{POS}$ human tumor cell lines.**

**(A)** Quantification in U266 or MM1.s in resting conditions or upon 48 hours IFNγ stimulation of soluble and membrane bound galectin-9 (N=2/3), Annexin V (N=6), CEACAM (N=3) and HMGB1 (N=2/3) (ligands of TIM-3; upper panels); CD48 (ligand of 2B4; bottom left; N=5) and HLA-DR (N=6) and FGL-1 (N=2/3) (ligands of LAG-3; bottom right). (**B**) Representative histograms showing membrane bound Galectin-9 (Galectin 9 – surface), CD48, HLA-DR, Annexin V and CEACAM expression in MM1.s and U266 unstimulated (light grey histograms) and 48h after IFNγ stimulation (dark grey histograms). Unstained cells (white histograms) are shown as control. (**C**) Schematic representations of lentiviral vectors encoding for the NY-ESO-1 antigen under hPGK promoter and ΔLNGFR selection marker under mCMV promoter [LV NY-ESO-1 (hPGK) ΔLNGFR (mCMV), upper panel] or encoding for HLA-A2 under hPGK promoter [LV (hPGK) HLA-A2), middle panel] or encoding for the firefly luciferase under hPGK promoter and green fluorescent protein under mCMV promoter [LV luc (hPGK) GFP (mCMV), lower panel]. (**D**) Representative plots of MM1.s engineered to express luciferase, HLA-A2 and NY-ESO-1 (upper panel) or MM1.s wt cells (bottom panel). In panel A, mean ± SEM from 2-6 technical replicates are shown, with the exception of membrane Galectin-9 (single replicate).

## Supplementary Figure 4

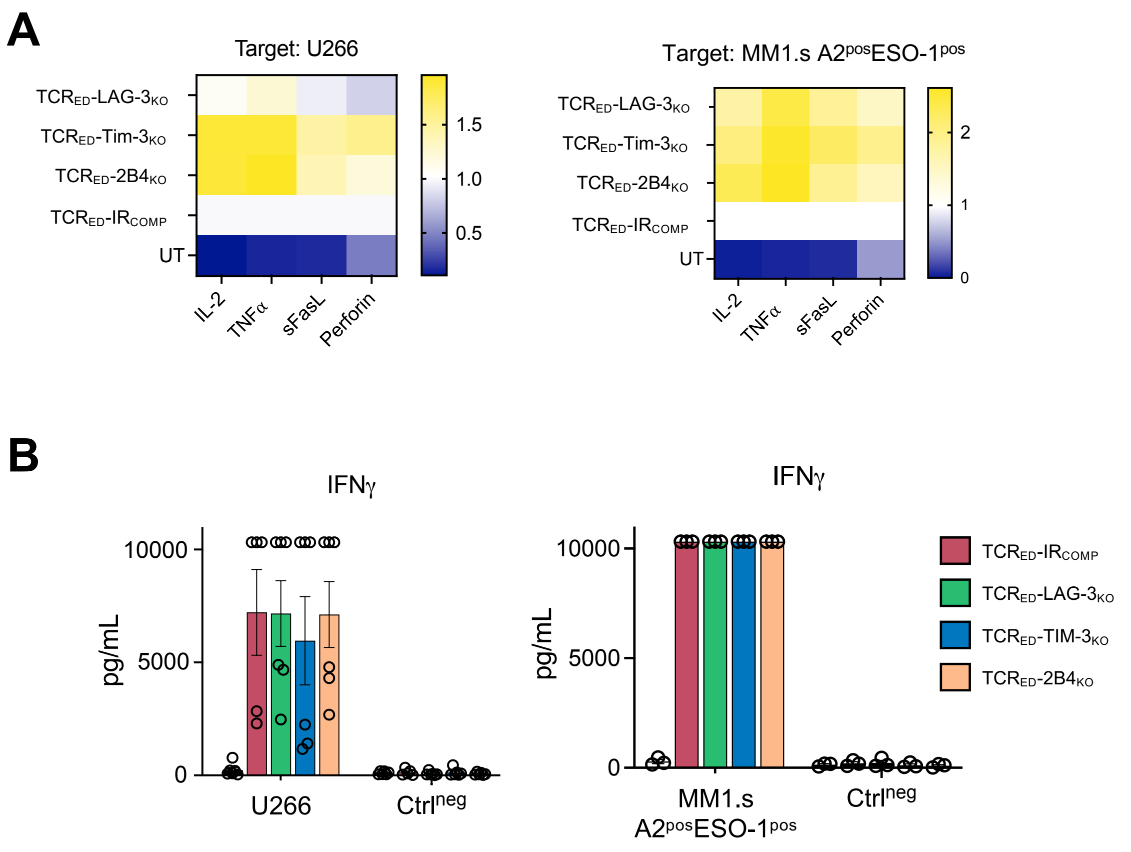


**Supplementary Figure 4. TCR$_{ED}$-IR$_{KO}$ T cells produce higher amount of effector molecules than TCR$_{ED}$-IR$_{COMP}$ T cells.**

(**A**) Heatmap showing the ratio of secreted effector molecules produced by TCR$_{ED}$-IR$_{KO}$ over TCR$_{ED}$-IR$_{COMP}$ cells upon 24 hours co-culture with U266 (left panel) or HLA-A2$^{pos}$ESO-1$^{pos}$ MM1s target cells (right panel). Data are shown as mean ± SEM of at least 3 biological replicates. (**B**) Quantification of IFNγ produced by TCR$_{ED}$-IR$_{COMP}$ (red bars), TCR$_{ED}$-LAG-3$_{KO}$ (green bars), TCR$_{ED}$-TIM-3$_{KO}$ (blue bars), and TCR$_{ED}$-2B4$_{KO}$ (light orange bars) T cells upon exposure to HLA-A2$^{pos}$ NY-ESO$^{pos}$ U266 cells (left panel; N=6) or MM1.s A2$^{pos}$ ESO-1$^{pos}$ cells (right panel; N=3). Negative controls (Ctrl$^{neg}$) indicate exposure of effector cells to medium or wild-type MM1.s cells. Data are shown as mean ± SEM of 3-6 biological replicates.

# Supplementary Figure 5

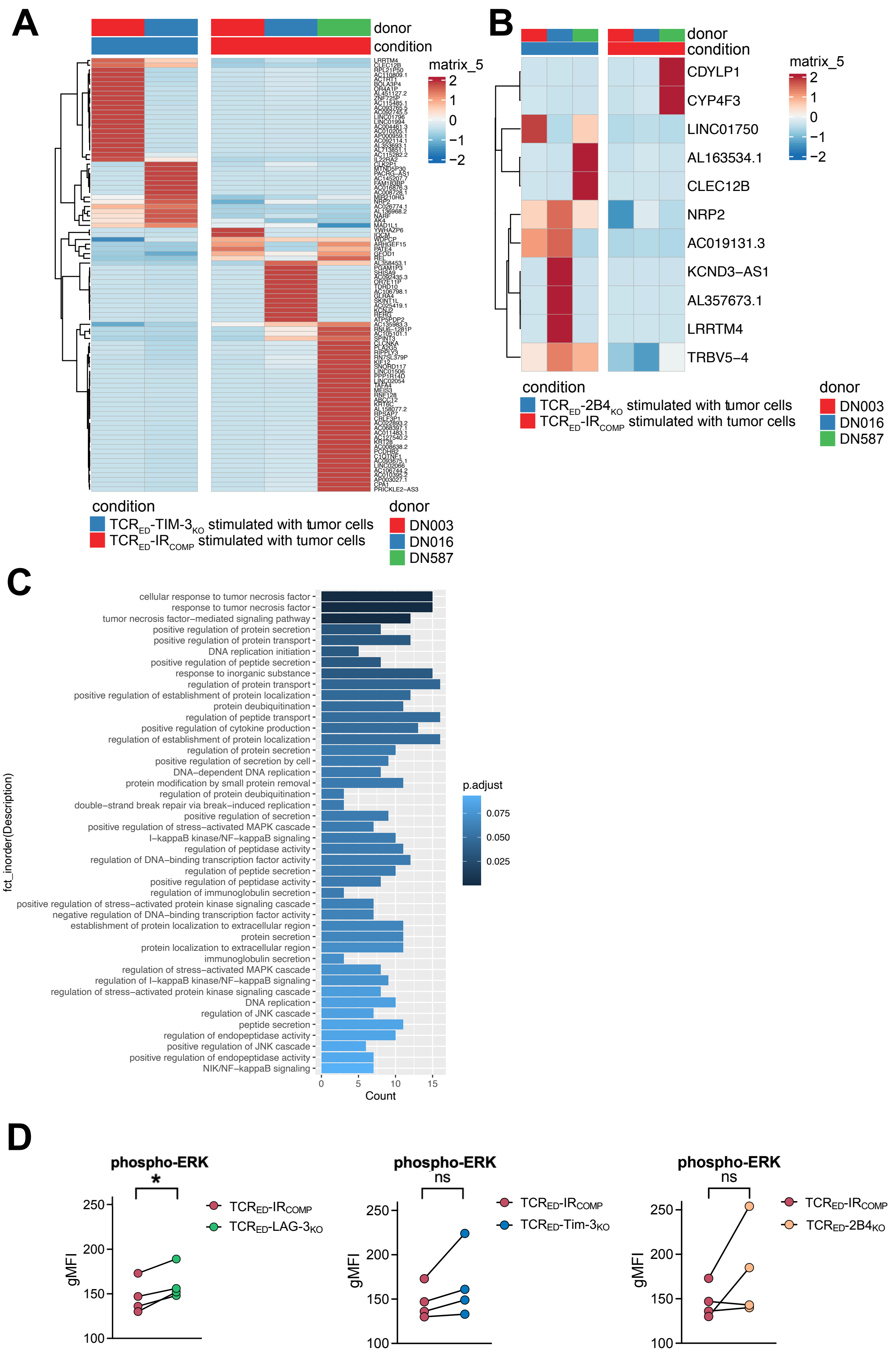

**Supplementary Figure 5. Gene expression analysis reveals specific transcriptomic changes in $TCR_{ED}$-$IR_{KO}$ T cells.**

Vulcano plots showing differentially expressed genes (DEGs) in $TCR_{ED}$-TIM-$3_{KO}$ **(A)** and $TCR_{ED}$-$2B4_{KO}$ **(B)** T cells upon stimulation with MM1.s $A2^{pos}$ ESO-$1^{pos}$ cancer cells. (**C**) Gene ontology (biological process) of the 30 uniquely DEGs in $TCR_{ED}$-$2B4_{KO}$ T cells upon stimulation with cancer cells. (**D**) Geometric mean fluorescence intensity (gMFI) of phospho-ERK measured by flow cytometry on $TCR_{ED}$-$IR_{KO}$ and $TCR_{ED}$-$IR_{COMP}$ cells 8 minutes after TCR triggering. Data obtained from 3 (panels A-C) and 4 (panel D) biological replicates are shown. *: p value <0.05; two-tailed paired t-test.

## Supplementary Figure 6

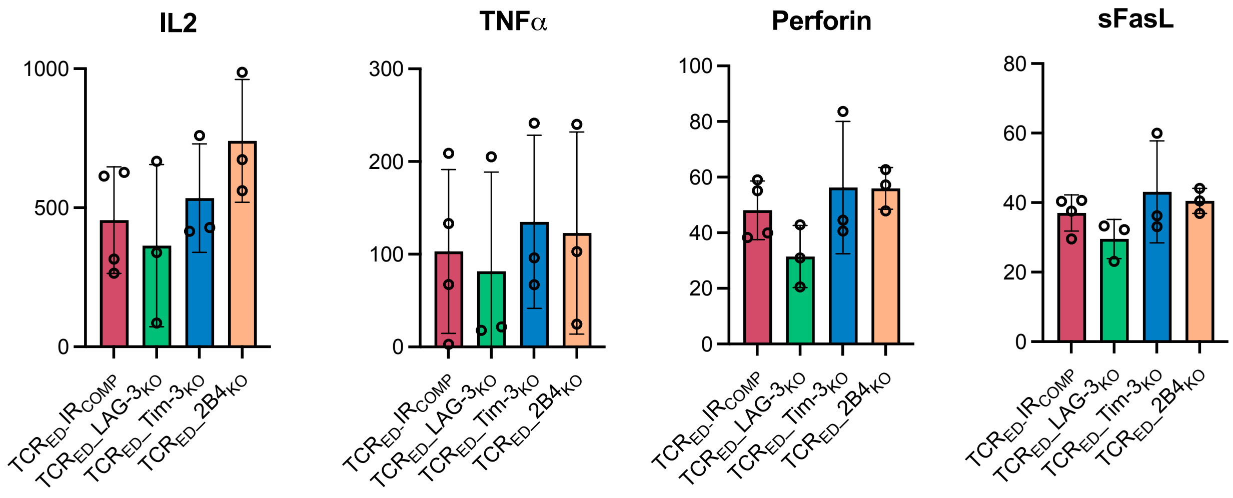


**Supplementary Figure 6. Similar cytokine secretion profile in TCR$_{ED}$-IR$_{KO}$ and TCR$_{ED}$-IR$_{COMP}$ T cells after chronic antigen stimulation**

TCR$_{ED}$-IR$_{COMP}$ and TCR$_{ED}$-IR$_{KO}$ cells were daily stimulated with MM1.s A2$^{pos}$ESO-1$^{pos}$ cells as described in Figure 4A. Quantification of IL-2, TNFα, perforin, and sFasL produced by TCR$_{ED}$-IR$_{COMP}$ (red bars; N=4), TCR$_{ED}$-LAG-3$_{KO}$ (green bars; N=3), TCR$_{ED}$-TIM-3$_{KO}$ (blue bars; N=3), and TCR$_{ED}$-2B4$_{KO}$ (light orange bars; N=3) upon exposure to HLA-A2$^{pos}$ NY-ESO$^{pos}$ MM1.s A2$^{pos}$ESO-1$^{pos}$ cells is shown. Data were obtained from 3-4 biological replicates.

## Supplementary Figure 7

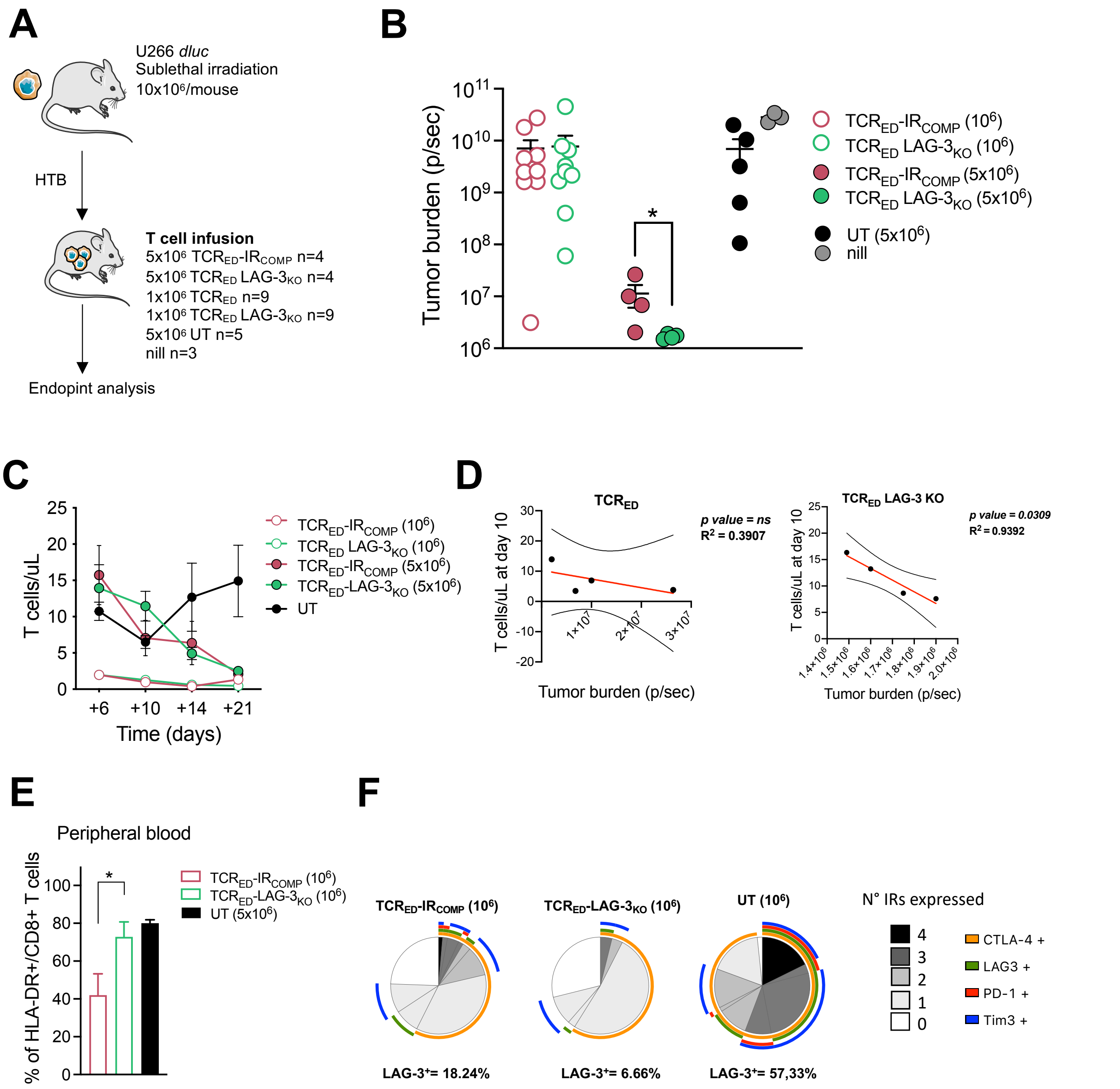

**Supplementary Figure 7. LAG-$3_{KO}$ cells prevents the up-regulation of IRs in $TCR_{ED}$ T cells *in vivo.***

(**A**) Schematic representation of *in vivo* high tumor burden (HTB) multiple myeloma model (U266). Tumor burden measured by total body bioluminescence, BLI (**B**) and absolute numbers of circulating human T cells (**C**) in mice treated with a low dose (empty circles) or a high dose (filled circles) of $TCR_{ED}$-$IR_{COMP}$ (red) or $TCR_{ED}$-LAG-$3_{KO}$ (green) cells or injected with a high dose of unmanipulated cells (UT, black) or left untreated (nil, gray). (**D**) Linear regression analysis of tumor burden and number of circulating T cells in treated mice. (**E**) Frequencies of activated (HLA-$DR^{+}$) T cells in the peripheral blood of mice treated with a low dose of $TCR_{ED}$-$IR_{COMP}$ (red) or $TCR_{ED}$-LAG-$3_{KO}$ (green) or UT cells (black). (**F**) Inhibitory receptors expression in T cells infiltrating the bone marrow of mice treated with low doses of $TCR_{ED}$-$IR_{COMP}$ or $TCR_{ED}$-LAG-$3_{KO}$ T cells or injected with UT cells. The slices in grayscale within the pie indicate the total number of co-expressed inhibitory receptors, while the external pie arches indicate the specific inhibitory receptor expressed. N. of treated and analyzed animals are shown in A.

## Supplementary Figure 8

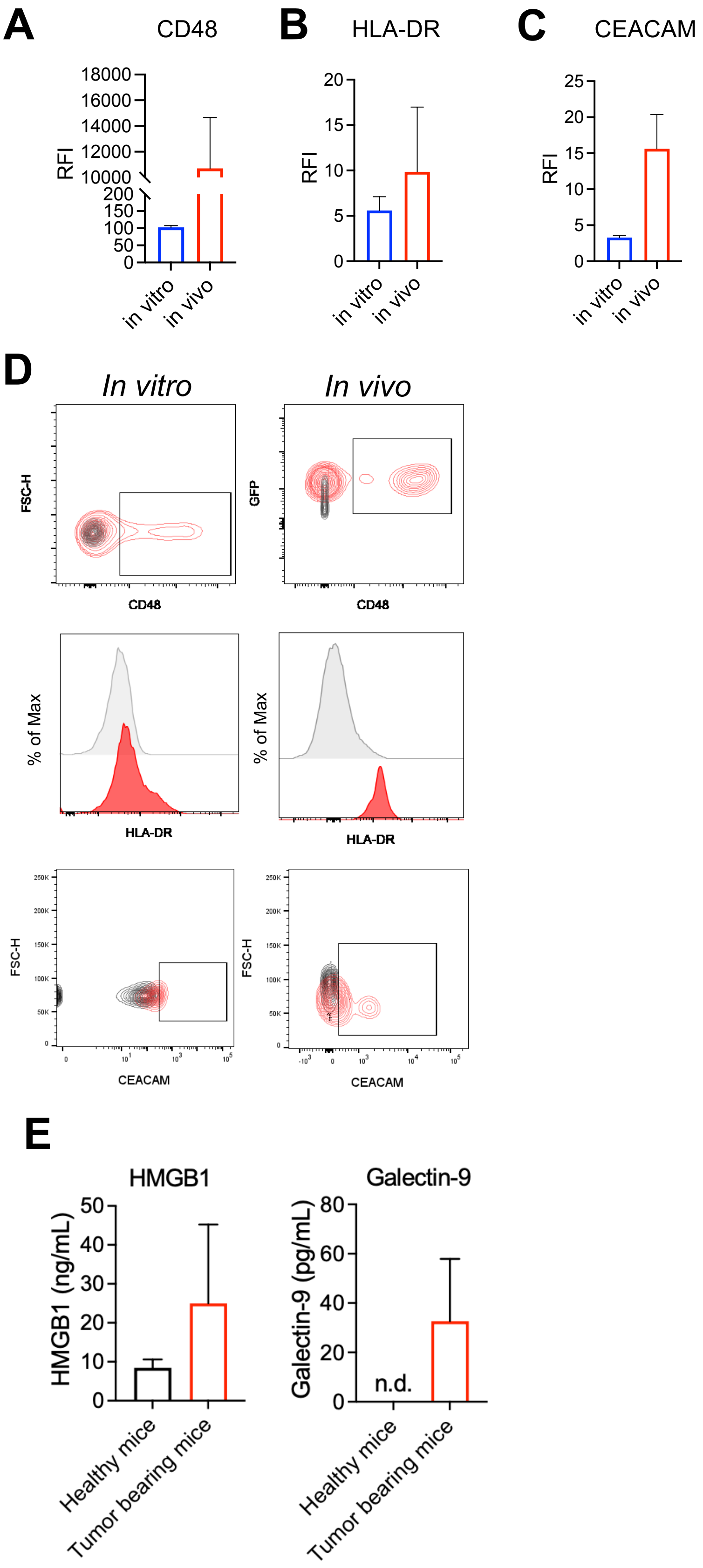

**Supplementary Figure 8. U266 cells upregulate IR ligands *in vivo*.**

Quantification of CD48 (**A**), HLA-DR (**B**) and CEACAM (**C**) expressed *in vitro* or *in vivo* on the cell surface of U266 cells (N=3). RFI (ratio of fluorescence intensity) = Mean of fluorescence intensity of stained cells / Mean of fluorescence intensity of unstained cells. (**D**) Representative plots of CD48, HLA-DR and CEACAM expression on U266 cells. Unstained samples are shown in grey while stained samples are shown in red. (**E**) Quantification of galectin-9 and HMGB1 in the serum of U266 injected mice (N=3) and healthy mice (N=2), at sacrifice.

## Supplementary Figure 9

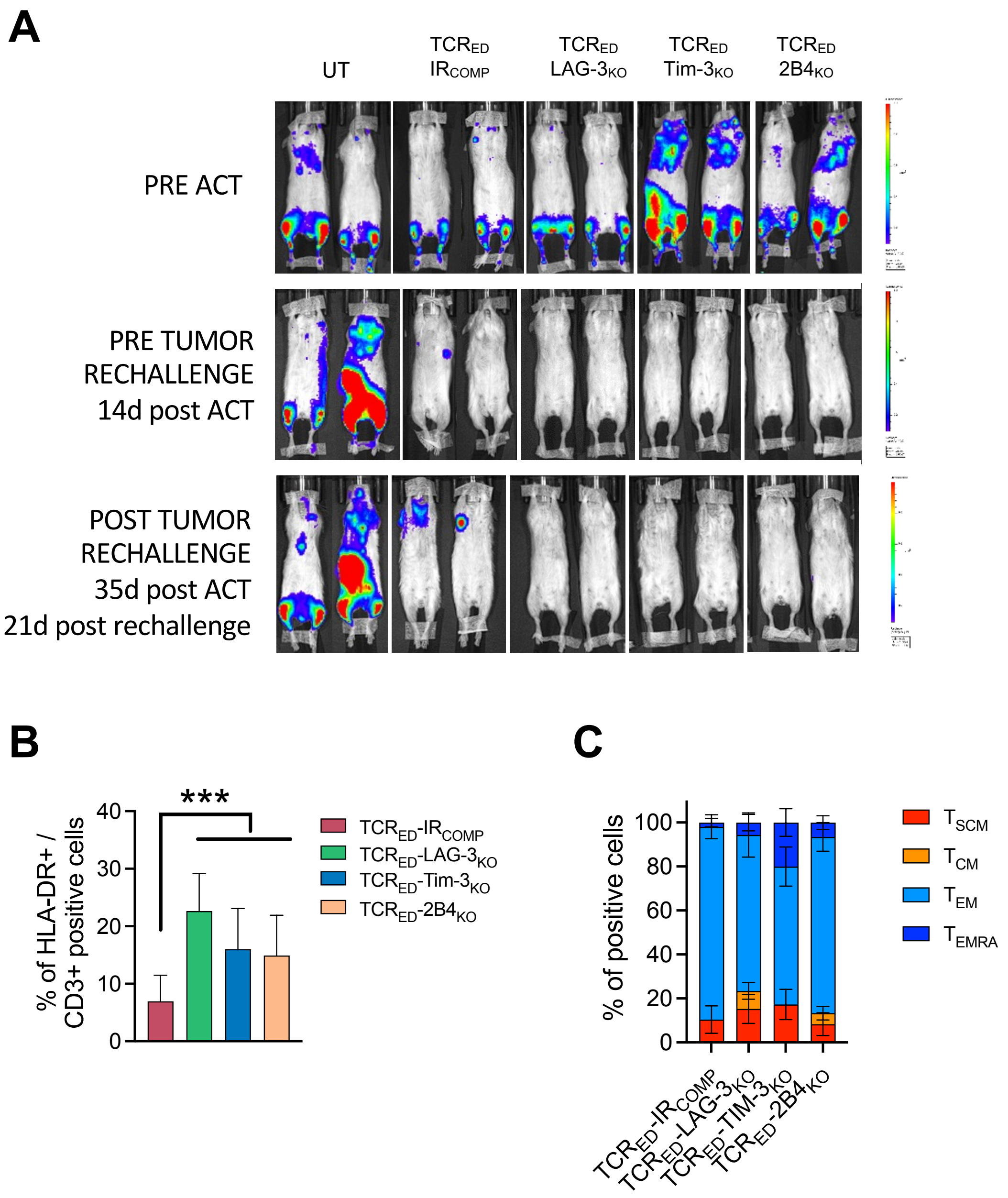

**Supplementary Figure 9. Activation and differentiation phenotypic profile of human T cells harvested in the rechallenge tumor model.**

**(A)** Bioluminescence images of representative mice injected with U266 cells according to **Figure** 5A. Mice were analysed before adoptive cell therapy with $TCR_{ED}$-$IR_{COMP}$ and $TCR_{ED}$-$IR_{KO}$ cells (upper panels), after adoptive cell therapy, but before rechallenge with U266 cells (middle panels) and after rechallenge with the tumor. (**B**) Frequencies of activated (HLA-$DR^{+}$) $CD3^{+}$ T cells in the spleen at sacrifice after second tumor challenge. (**C**) Frequencies of stem memory T cells ($T_{SCM}$), central memory ($T_{CM}$), effector memory ($T_{EM}$) and terminally differentiated effector cells ($T_{EMRA}$) in human CD3+ T cells in the spleen at sacrifice after second tumor challenge. Data are shown as mean $\pm$ SEM. N=6 for $TCR_{ED}$-$IR_{COMP}$ (red), $TCR_{ED}$-LAG-$3_{KO}$ (green), $TCR_{ED}$-TIM-$3_{KO}$ (blue); N=5 $TCR_{ED}$-$2B4_{KO}$ (light orange).
