# Supplementary material for: TIM-3, LAG-3, or 2B4 gene disruptions increase the anti-tumor response of engineered T cells
Source: Front Immunol. 2024 Feb 29;15:1315283. doi: 10.3389/fimmu.2024.1315283 (PMC10953820; doi:10.3389/fimmu.2024.1315283)

Supplementary Figure 1

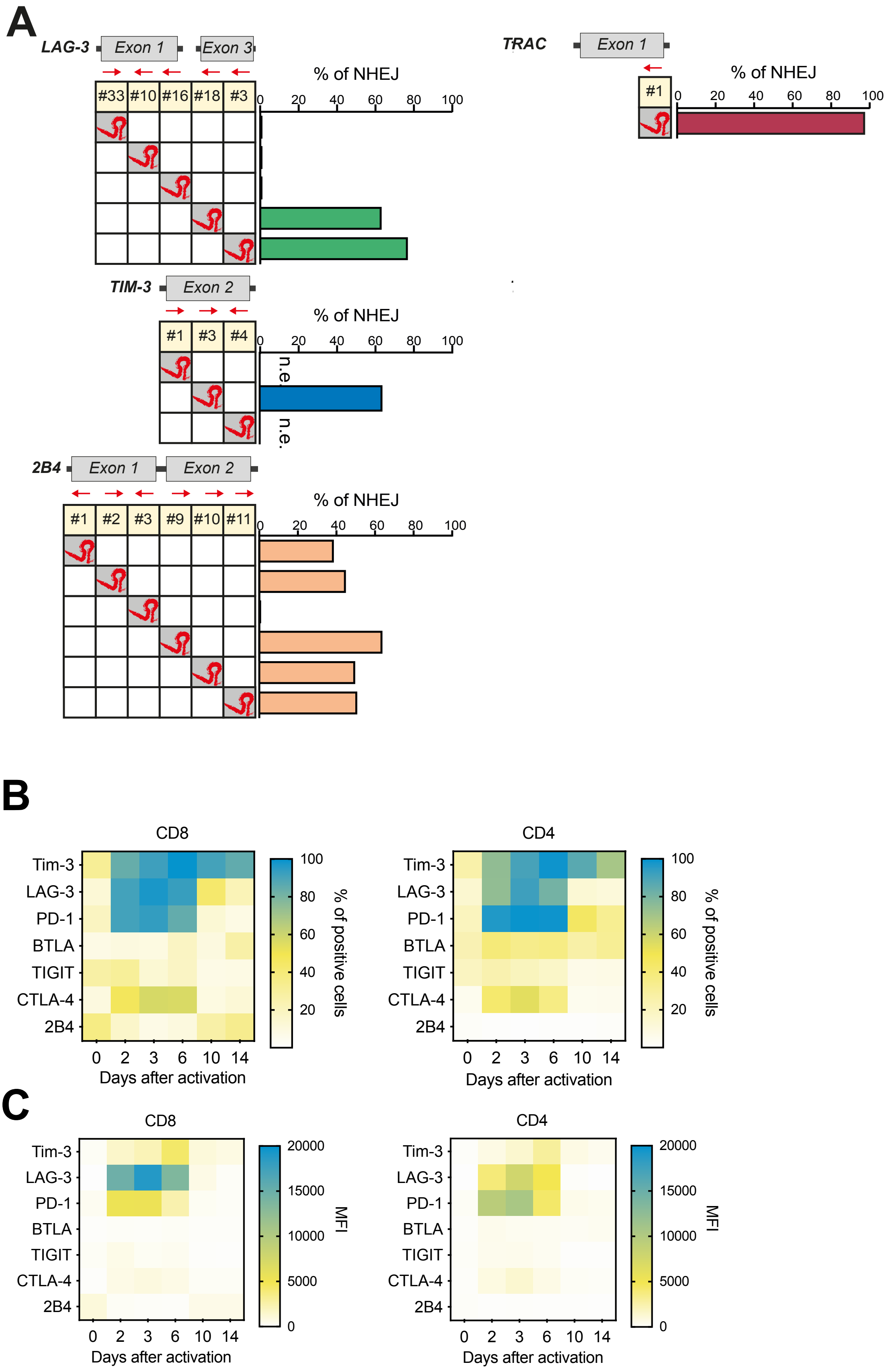

Supplementary Figure 2

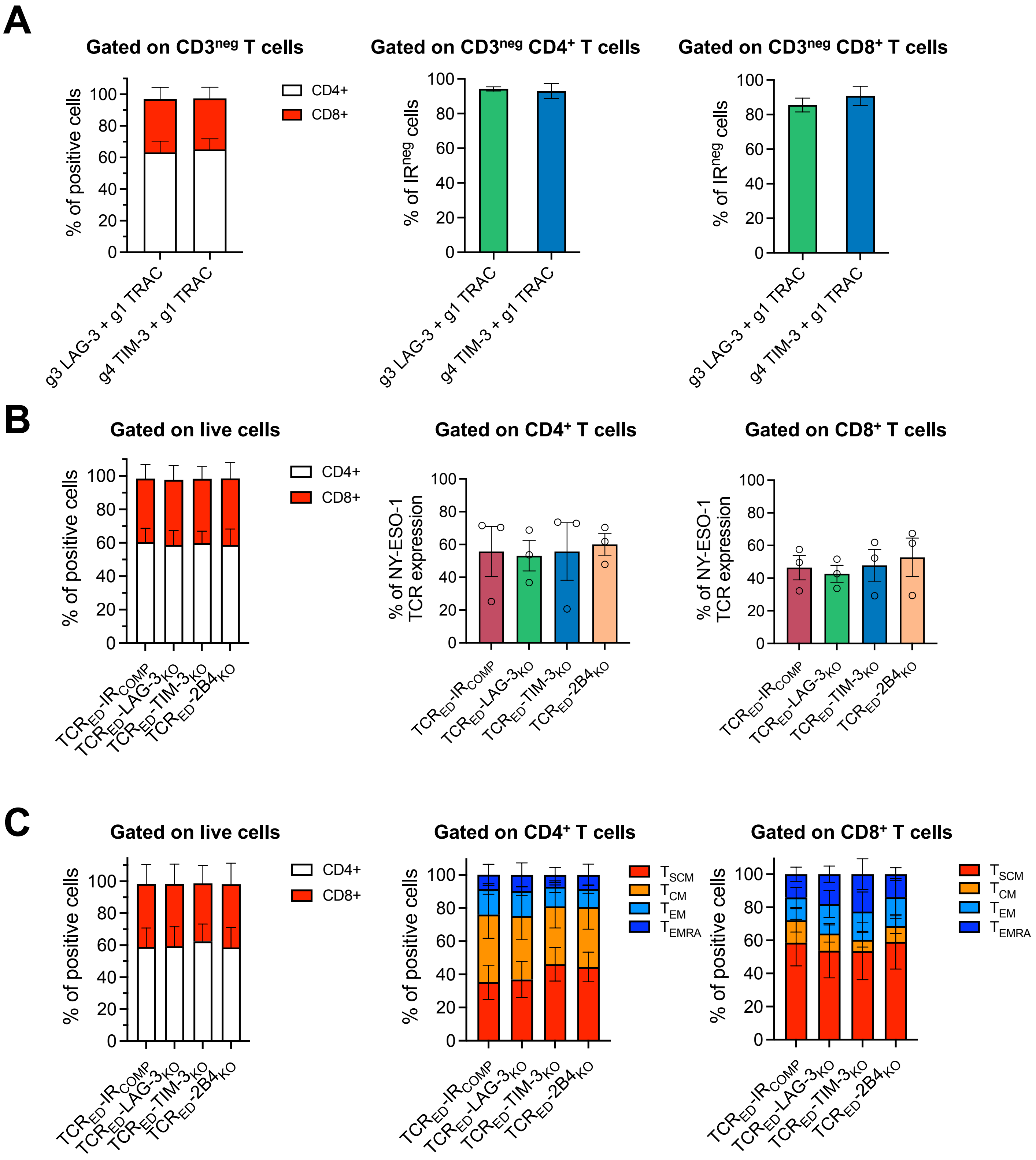

D

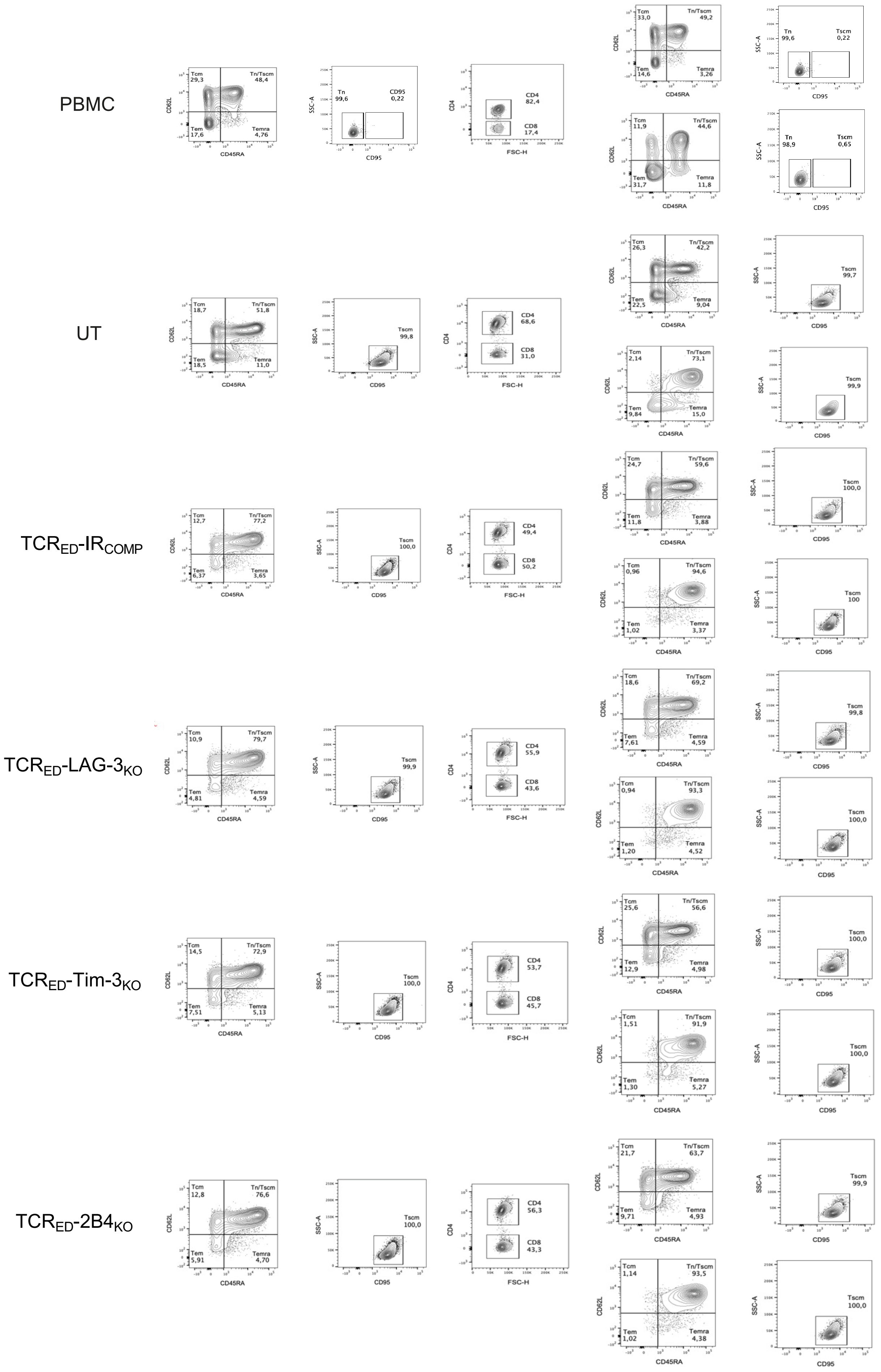
PBMC
UT
TCR$_{ED}$-IR$_{COMP}$
TCR$_{ED}$-LAG-3$_{KO}$
TCR$_{ED}$-Tim-3$_{KO}$
TCR$_{ED}$-2B4$_{KO}$
CD62L
CD45RA
SSC-A
CD95
CD4
FSC-H
Tcm 29,3 Tn/Tscm 48,4 Tem 17,6 Temra 4,76
Tn 99,6 CD95 0,22
CD4 82,4 CD8 17,4
Tcm 33,0 Tn/Tscm 49,2 Tem 14,6 Temra 3,26
Tn 99,6 Tscm 0,22
Tcm 11,9 Tn/Tscm 44,6 Tem 31,7 Temra 11,8
Tn 98,9 Tscm 0,65
Tcm 18,7 Tn/Tscm 51,8 Tem 18,5 Temra 11,0
Tscm 99,8
CD4 68,6 CD8 31,0
Tcm 26,3 Tn/Tscm 42,2 Tem 22,5 Temra 9,04
Tscm 99,7
Tcm 2,14 Tn/Tscm 73,1 Tem 9,84 Temra 15,0
Tscm 99,9
Tcm 12,7 Tn/Tscm 77,2 Tem 6,37 Temra 3,65
Tscm 100,0
CD4 49,4 CD8 50,2
Tcm 24,7 Tn/Tscm 59,6 Tem 11,8 Temra 3,88
Tscm 100,0
Tcm 0,96 Tn/Tscm 94,6 Tem 1,02 Temra 3,37
Tscm 100
Tcm 10,9 Tn/Tscm 79,7 Tem 4,81 Temra 4,59
Tscm 99,9
CD4 55,9 CD8 43,6
Tcm 18,6 Tn/Tscm 69,2 Tem 7,61 Temra 4,59
Tscm 99,8
Tcm 0,94 Tn/Tscm 93,3 Tem 1,20 Temra 4,52
Tscm 100,0
Tcm 14,5 Tn/Tscm 72,9 Tem 7,51 Temra 5,13
Tscm 100,0
CD4 53,7 CD8 45,7
Tcm 25,6 Tn/Tscm 56,6 Tem 12,9 Temra 4,98
Tscm 100,0
Tcm 1,51 Tn/Tscm 91,9 Tem 1,30 Temra 5,27
Tscm 100,0
Tcm 12,8 Tn/Tscm 76,6 Tem 5,91 Temra 4,70
Tscm 100,0
CD4 56,3 CD8 43,3
Tcm 21,7 Tn/Tscm 63,7 Tem 9,71 Temra 4,93
Tscm 99,9
Tcm 1,14 Tn/Tscm 93,5 Tem 1,02 Temra 4,38
Tscm 100,0

Supplementary Figure 3

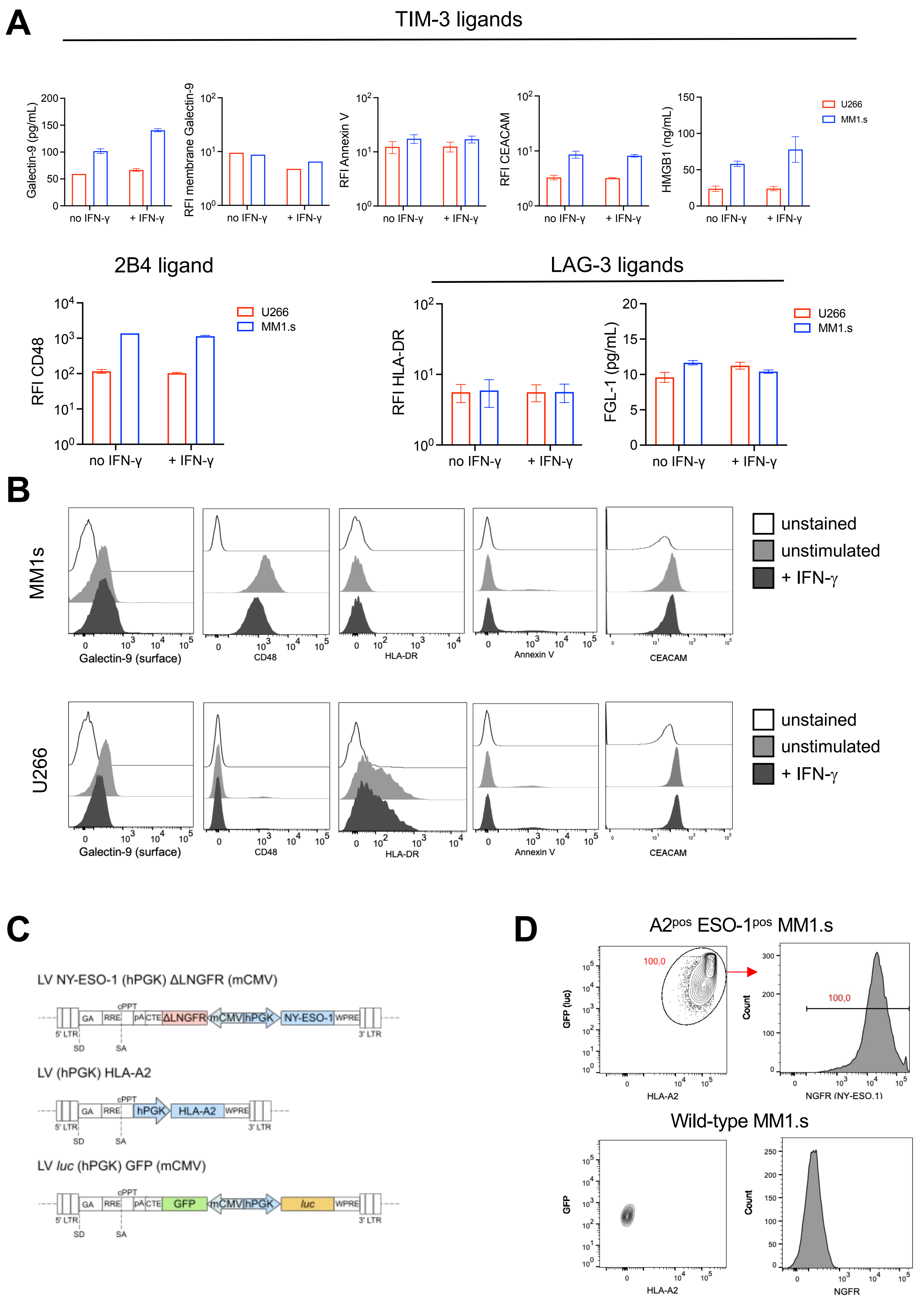

Supplementary Figure 4

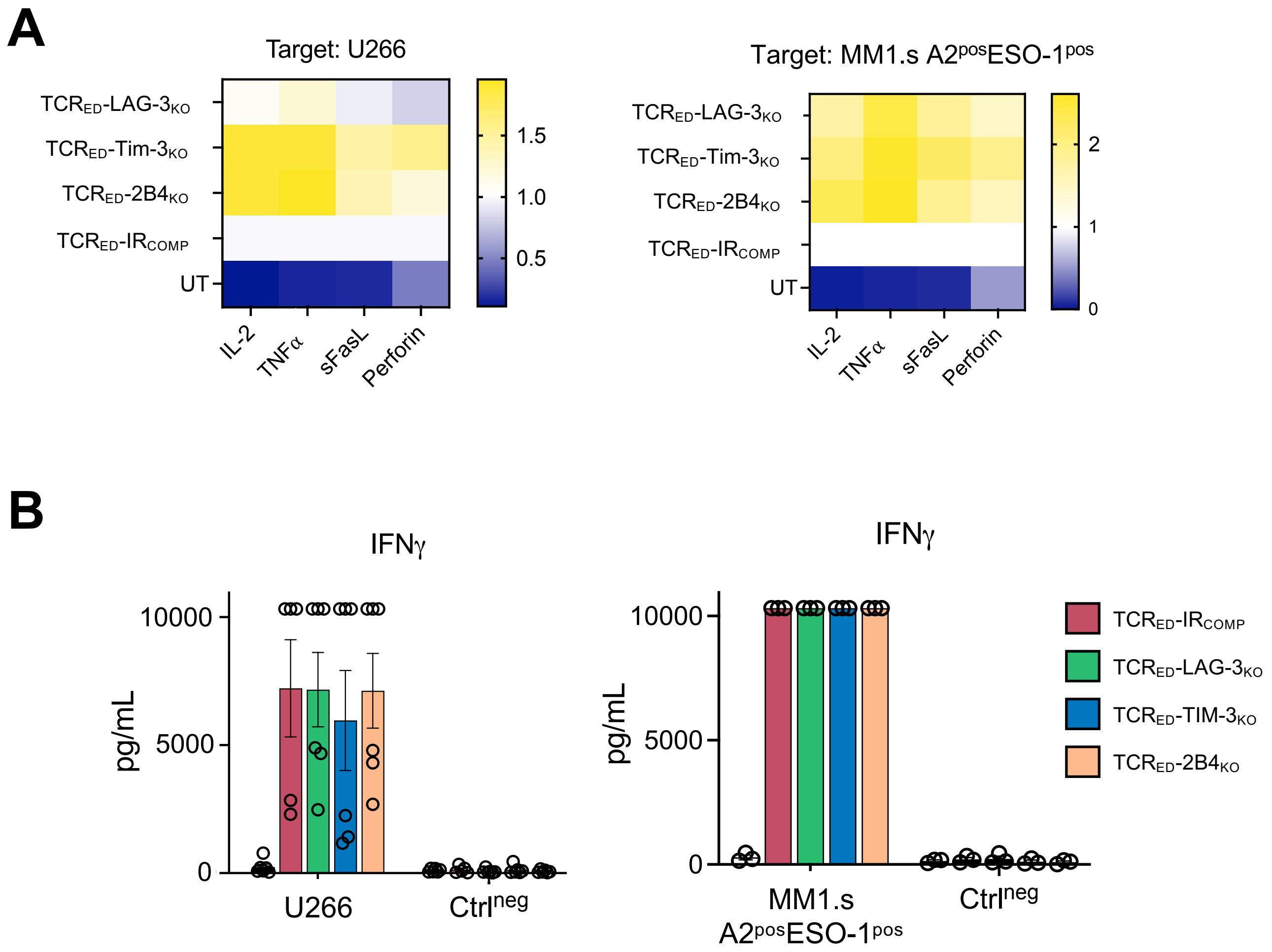

Supplementary Figure 5

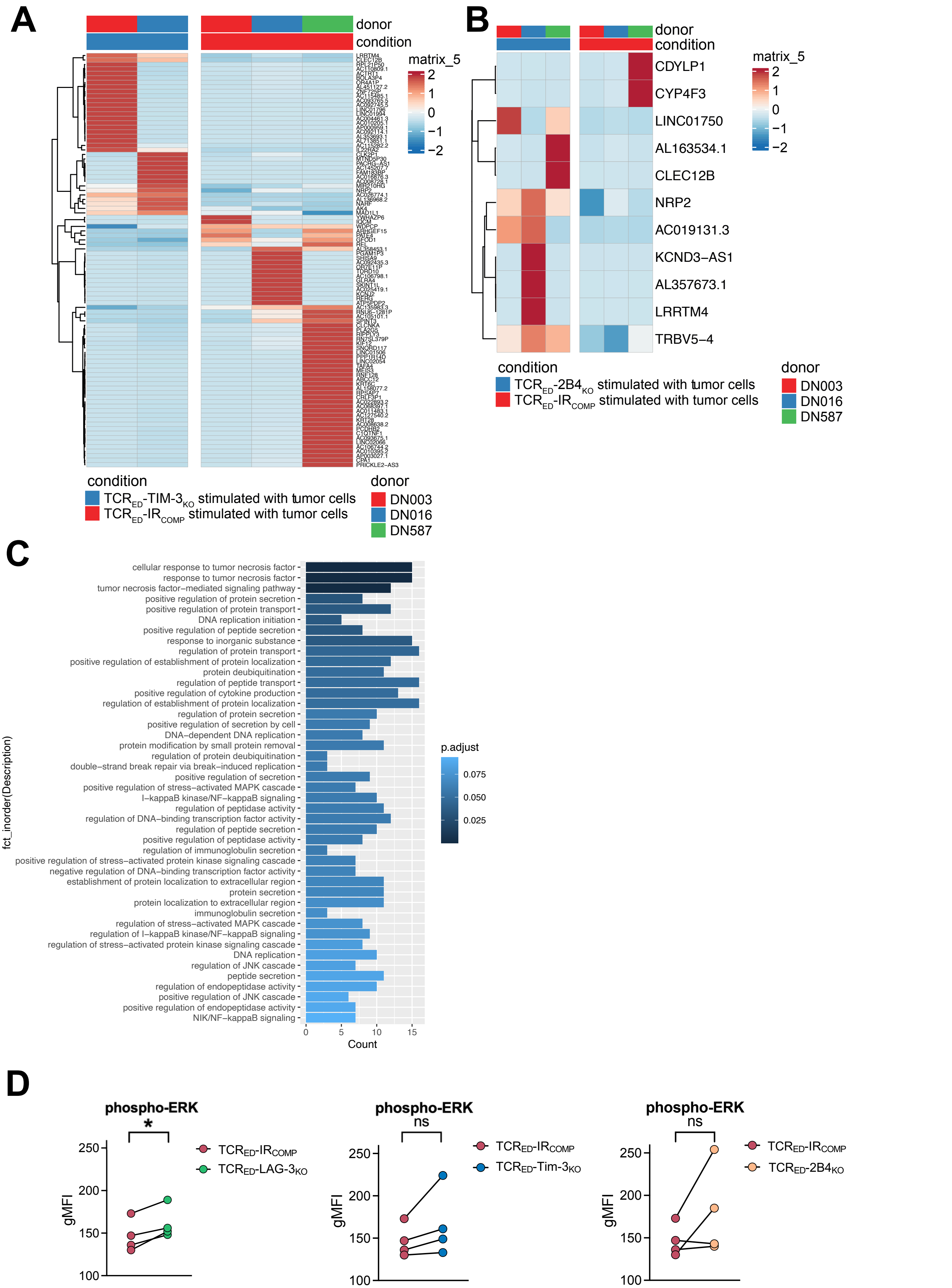

Supplementary Figure 6

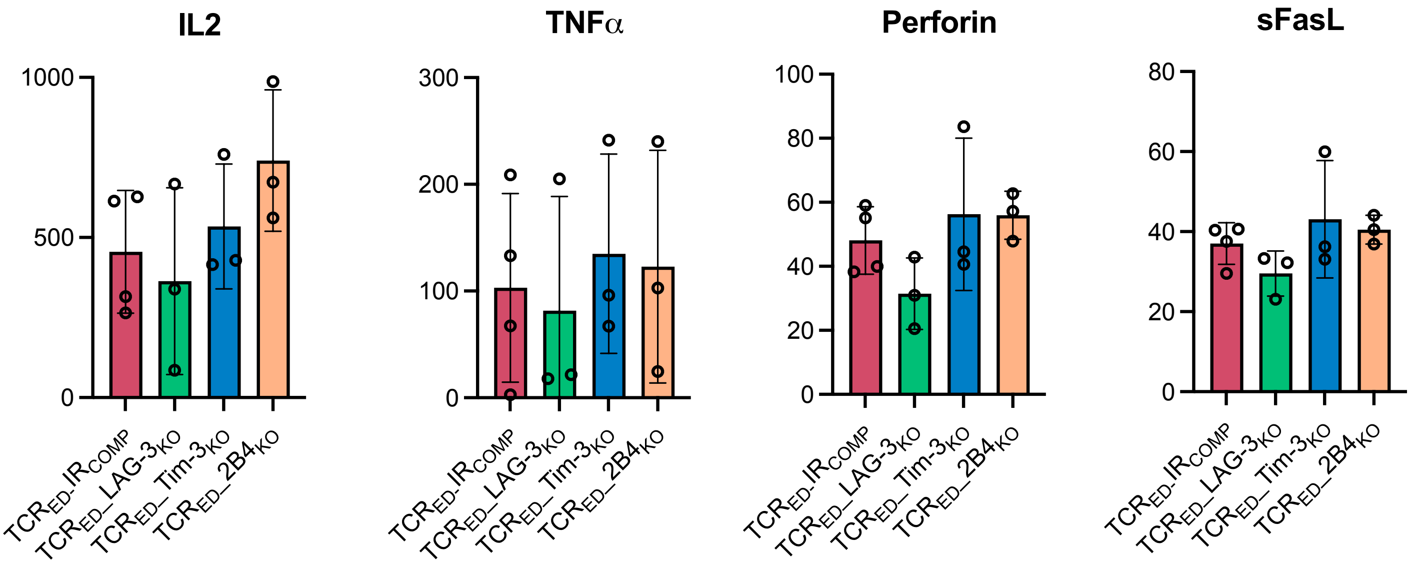

Supplementary Figure 7

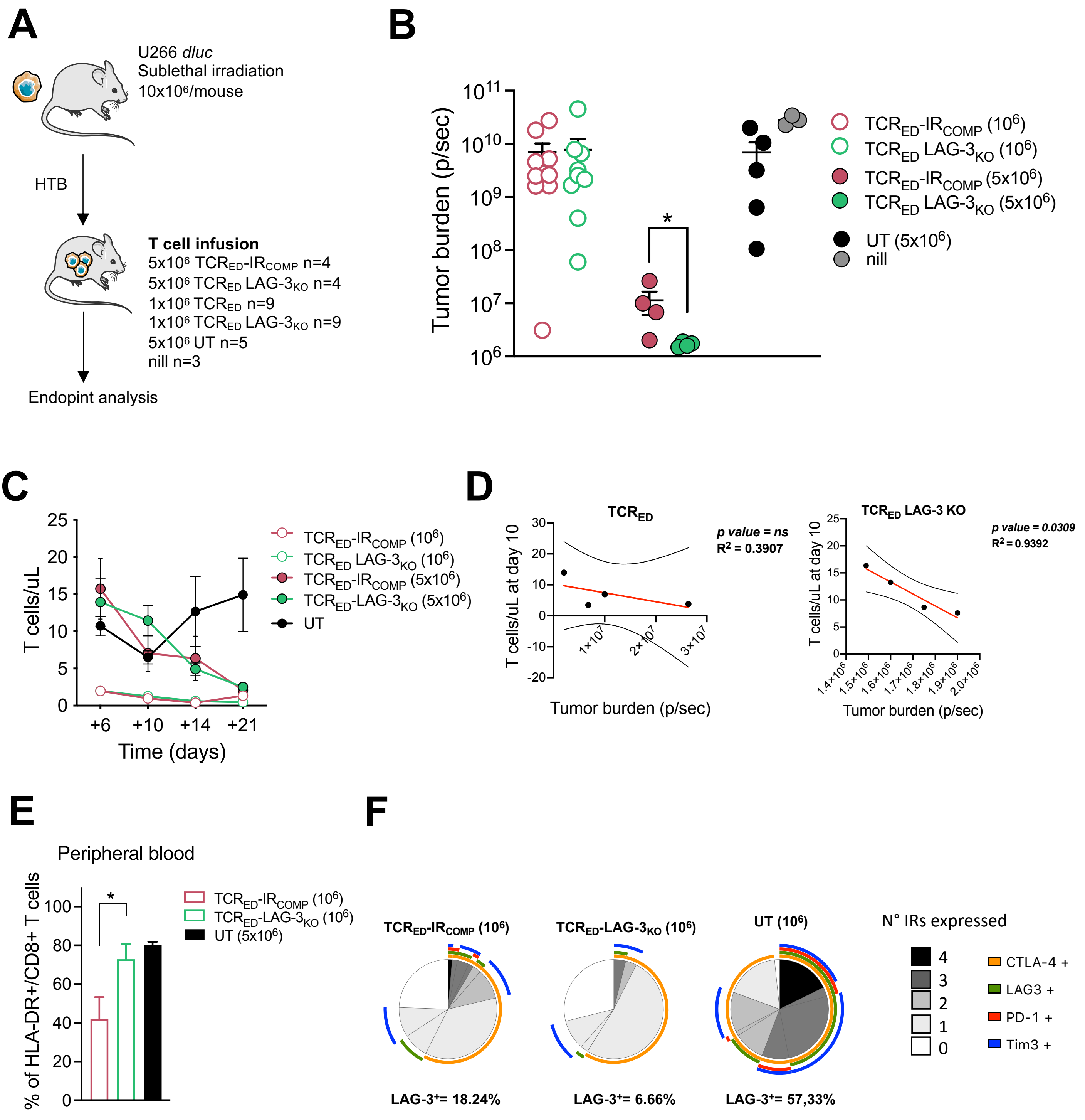

Supplementary Figure 8

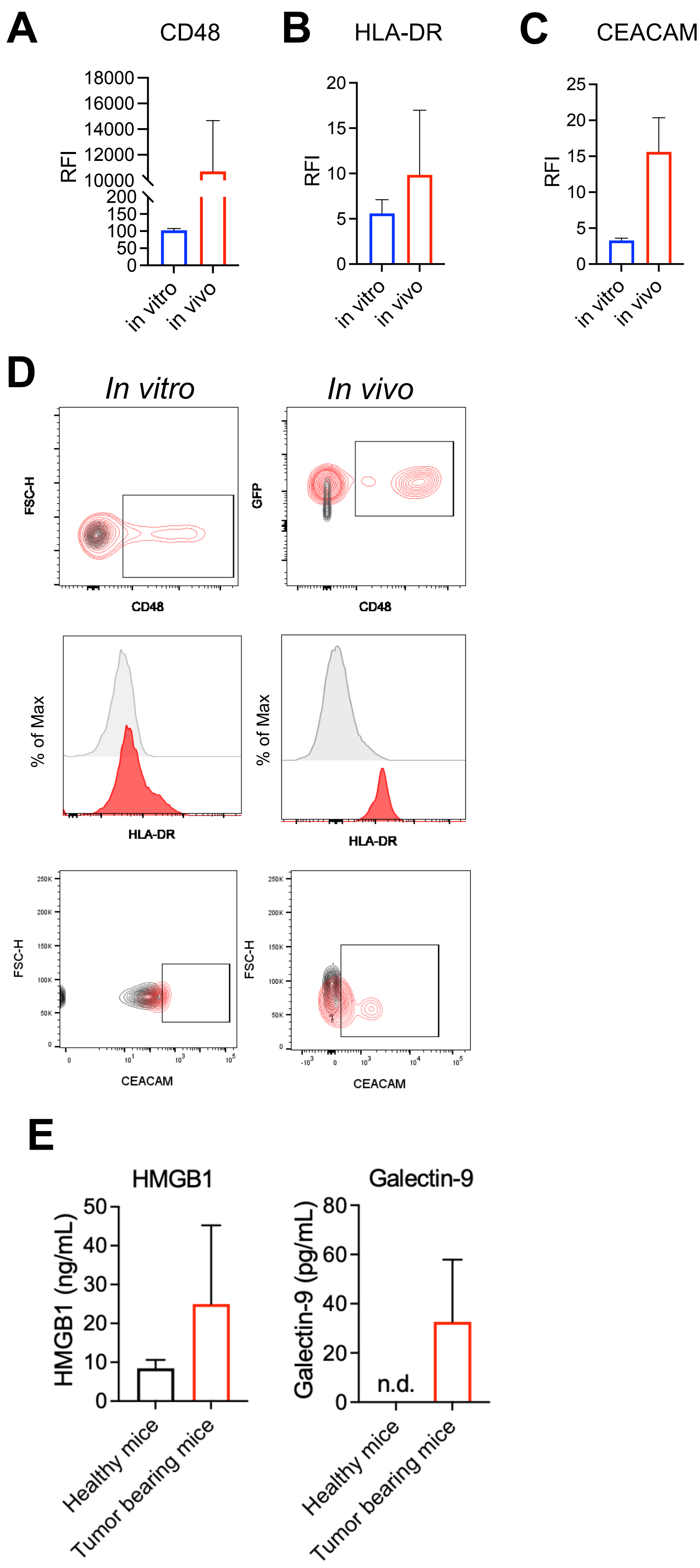

Supplementary Figure 9

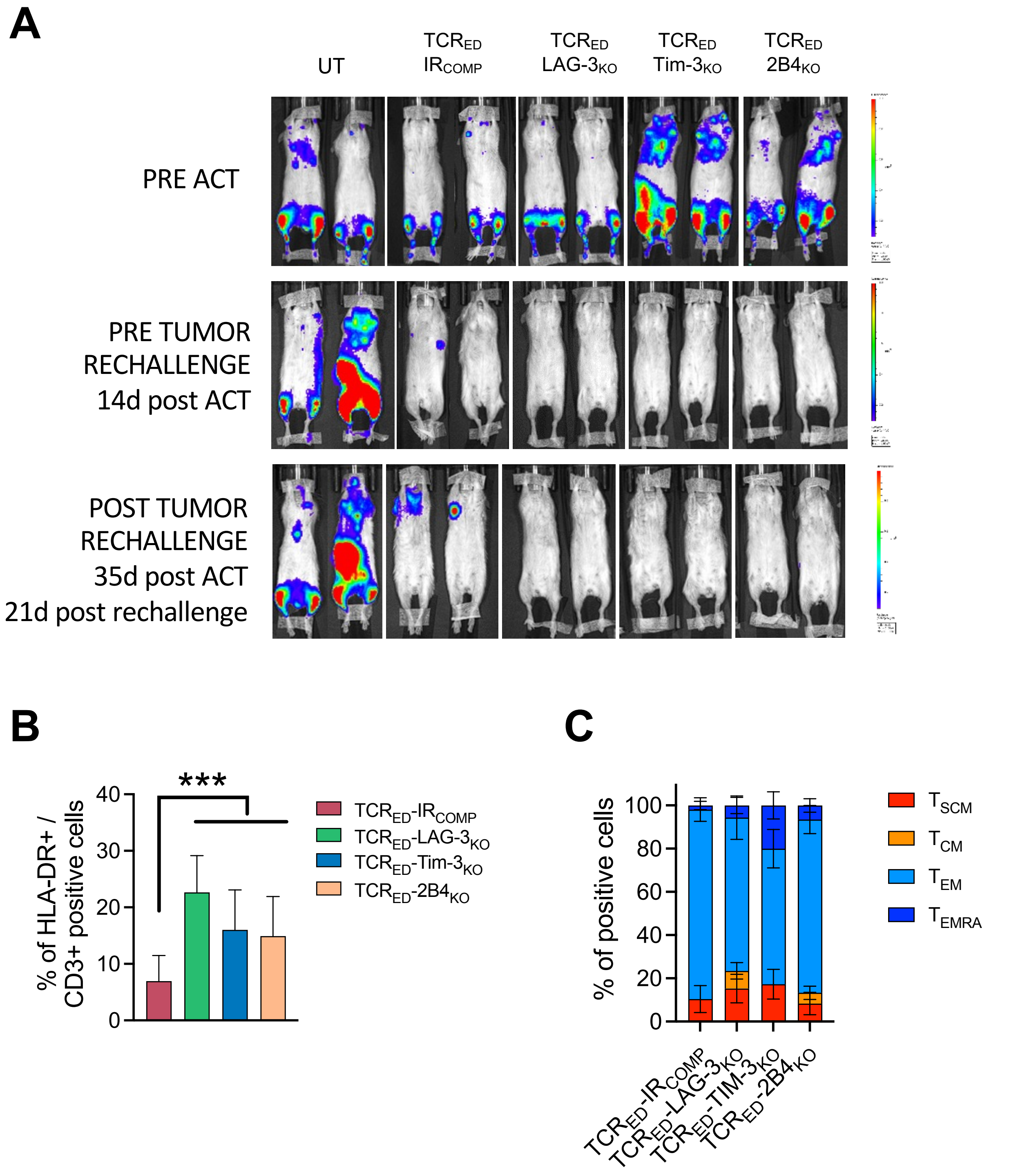

Supplement: Supplementary file 2 [file DataSheet_2.pdf]
